# A harmonized atlas of mouse spinal cord cell types and their spatial organization

**DOI:** 10.1038/s41467-021-25125-1

**Published:** 2021-09-29

**Authors:** Daniel E. Russ, Ryan B. Patterson Cross, Li Li, Stephanie C. Koch, Kaya J. E. Matson, Archana Yadav, Mor R. Alkaslasi, Dylan I. Lee, Claire E. Le Pichon, Vilas Menon, Ariel J. Levine

**Affiliations:** 1 Division of Cancer Epidemiology and Genetics, Data Science Research Group, National Cancer Institute, NIH, Rockville, MD USA; 2 Spinal Circuits and Plasticity Unit, National Institute of Neurological Disorders and Stroke, NIH, Bethesda, MD USA; 3 Department of Neuroscience, Physiology and Pharmacology, Division of Biosciences, University College London, London, UK; 4 Department of Neurology, Center for Translational and Computational Neuroimmunology, Columbia University, New York, NY USA; 5 Eunice Kennedy Shriver National Institute of Child Health and Human Development, NIH, Bethesda, MD USA; 6 Department of Neuroscience, Brown University, Providence, RI USA

**Keywords:** Transcriptomics, Cellular neuroscience, Molecular neuroscience, Spinal cord

## Abstract

Single-cell RNA sequencing data can unveil the molecular diversity of cell types. Cell type atlases of the mouse spinal cord have been published in recent years but have not been integrated together. Here, we generate an atlas of spinal cell types based on single-cell transcriptomic data, unifying the available datasets into a common reference framework. We report a hierarchical structure of postnatal cell type relationships, with location providing the highest level of organization, then neurotransmitter status, family, and finally, dozens of refined populations. We validate a combinatorial marker code for each neuronal cell type and map their spatial distributions in the adult spinal cord. We also show complex lineage relationships among postnatal cell types. Additionally, we develop an open-source cell type classifier, SeqSeek, to facilitate the standardization of cell type identification. This work provides an integrated view of spinal cell types, their gene expression signatures, and their molecular organization.

## Abstract (web summary)

Single-cell profiling has led to the identification of diverse cell types. Here, the authors generate a harmonized cell atlas of the mouse post-natal spinal cord. They also provide spatial analysis of the distribution of the identified cell types and an open-source cell type classifier.

## Introduction

A revolution in single-cell sequencing technologies is transforming many fields of biology. By sequencing the RNA/cDNA or open chromatin from many individual cells and using computational analysis to identify shared patterns of gene expression or epigenetic structure, we may simultaneously define cell types, characterize their molecular signatures, and track how each cell type in tissue changes in different biological conditions such as development and disease. Within the central nervous system, this approach may also reveal the molecular basis of the impressive levels of neuronal diversity, can provide marker genes for developing genetic tools to manipulate neuronal function, and may help to reveal the cellular basis of behavior.

In the postnatal mouse spinal cord, there have been several papers profiling single-cell RNA expression that, combined, cover a range of biological parameters, including age, tissue region, developmental lineage, and circuit features^1–11^. These studies provide a powerful and multi-faceted perspective on spinal cord cell types, yet despite this significant effort and a rich literature of spinal cord cell type characterization (see reviews^12–19^), there is still no consensus cell type atlas of the spinal cord^20^. A major obstacle is the lack of accepted ground truth of cell types in this tissue that could form the basis of a reference atlas. Unfortunately, this challenge is compounded by the difficulty in comparing data between studies even when the same tissue types and techniques are used^3,5^. This is partly due to biological differences and technical limitations, but may also reflect particular analysis parameters and technical artifacts that conceal underlying similarities between these studies. Indeed, it is not clear whether the cell types from the original studies are comparable in their current forms, resulting in a fragmented set of incomplete and conflicting atlases for the spinal cord. Rather than being specific to the study of the spinal cord, these are among the grand challenges that scientists face as we re-discover the cells and tissues we study through the perspective of single-cell profiling^21^.

To begin to overcome these challenges within the mammalian central nervous system, we sought to establish a harmonized, validated atlas of postnatal spinal cord cell types that could reveal the organizing principles of spinal neuronal diversity and serve as a standard foundation for future work. We began by performing a merged and integrated analysis of the raw data from the first six publicly available postnatal spinal cord single-cell datasets. We clustered the cells and nuclei of this meta-dataset to reveal 15 non-neural and 69 neural cell types, thereby providing a cell type resolution and characterization that surpasses all prior studies, both in the depth of its detail and the breadth of general trends. By analyzing gene expression profiles across families of cell types, we created a combinatorial panel of dozens of marker genes and validated it with high-content in situ hybridization to characterize the spatial distribution and prevalence of each cell type in adult tissue. This work revealed striking differences between dorsal and ventral neuronal cell types, both in their cell-type relationships and molecular trends. Co-integration with embryonic cell types allowed us to infer putative lineage relationships for each postnatal cell type and uncovered complex convergent contributions from multiple lineages to many cell types. Finally, we tested a range of automated classification algorithms and identified a two-tiered model based on label transfer and neural networks as the best method for classifying spinal cord cell types. We now present SeqSeek, a web-based resource for querying this data by gene or cell type and for accessing automated classification algorithm of any spinal cord cell or nucleus from raw sequencing data.

## Results

### Merged analysis of spinal cord cells and nuclei

**Fig. 1:**
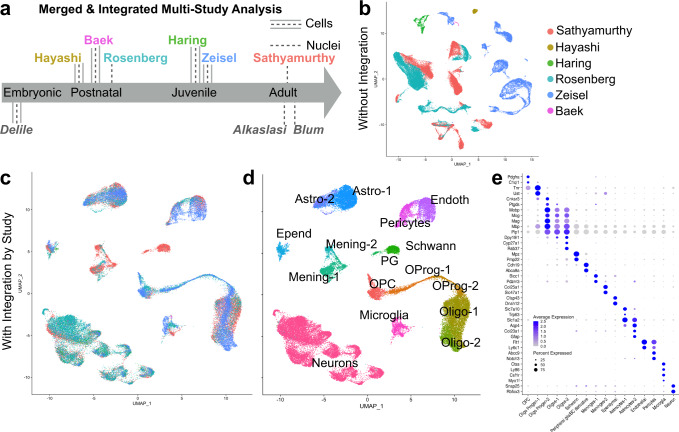
Integration of six independent studies on single cell spinal cord data reveals the major cell types of the spinal cord. **a** Summary of the datasets used in this study, including the studies that used single-cell/nucleus RNA sequencing to analyze postnatal mouse spinal cord cell types (colored names above the gray bar) and additional studies that were used for focused aspects of the analysis below the gray bar. The age and technique (cell or nuclei isolation) is represented for each study. **b** UMAP presentation of the 52,623 cells/nuclei in the final dataset, without integration and colored by the study of origin (colors in the legend). **c** UMAP presentation of the same 52,623 cells/nuclei in the final dataset, integrated by study and colored by the study of origin (same colors as in (**b**)). **d** UMAP presentation of the cells/nuclei in the merged dataset, integrated by study and colored by cell type. **e** Dot plot of the expression of marker genes for the major coarse cell types. Average expression for each cluster is shown by color intensity and the percent of cells/nuclei in each cluster that expressed each gene is shown by dot diameter.

We first created a merged dataset with over one hundred thousand cells and nuclei from the first six published studies of the postnatal mouse spinal cord^1–6^. These studies cover a range of biological and experimental parameters (Fig. 1a). To best compare the data from these studies, we began with the raw sequencing reads from each study and performed our own data processing with uniform methods and filters. All sequencing reads were aligned to a common genomic sequence that included both exons and introns and we used common, liberal filtering thresholds for inclusion (> 200 genes per cell/nucleus) and exclusion (<5% percent of genes from mitochondria). As a result, this merged dataset contains more cells and nuclei than were analyzed in the original studies and a uniform set of genes.

Our first major goal was to create a harmonized atlas of the major spinal cord cell types that are shared across these studies and we considered whether it would be possible to register different studies to each other and thereby identify a common set of cell types that would simply require the resolution of differences in nomenclature. To perform a direct comparison of the clusters between different studies, we used the merged data (with common threshold criteria and genes analyzed) and we focused on dorsal neurons that were commonly studied by Sathyamurthy et al., Haring et al., and Zeisel et al. For each study, we calculated the mean expression of each gene in each cluster and then analyzed the correlation in overall gene expression between the studies. When either all genes or the top 500 highly variable genes were analyzed, there were weak overall correlations and very few alignments between clusters from different studies (Supplementary Fig. 1a, b, d). We therefore concluded that the previously published atlases cannot simply be registered to each other to achieve a valid reference atlas. This is similar to previous reports which used correlation in gene expression between clusters to attempt to link cell types across studies and this approach yielded weak and/or incomplete correlations, even between studies in which the same sample age and tissue dissociation method were used^3,5^.

Next, we hypothesized that co-clustering cells and nuclei across all of the studies would provide an improved ability to relate cell types in one study to those in another. We performed dimensionality reduction using principal component analysis and visualized the cells and nuclei using Unifrom Manifold Approximation and Projection (UMAP) plots. Unfortunately, the cells or nuclei from each study were segregated from each other almost completely, indicating that the study of origin is a major source of variability in the dataset (Fig. 1b). This technical limitation obscured all cell type distinctions.

Finally, we used a recently developed integration method, implemented in the Seurat software package, to align the cells and nuclei across studies to reduce experimental sources of variability and reveal the core set of spinal cord cell types^22,23^. With this approach, the cells and nuclei from all six studies were spatially interposed in a UMAP visualization of principal component space (Fig. 1c) and separated into groupings that each expressed a panel of well-established cell type markers such as Snap25 (neurons), Mbp (oligodendrocytes), Aqp4 (astrocytes), and Ctss (microglia) (Fig. 1d, e). Moreover, the integration-adjusted gene expression values markedly improved the ability to identify relationships between the clusters of the original studies and also improved the top correlation score for each original cluster (Supplementary Fig. 1c, d).

With the integrated merged data, we performed preliminary clustering and removed low-quality clusters and doublets (see Methods) to obtain a final dataset of over fifty thousand cells and nuclei (Supplementary Fig. 2a, e). The majority of these cells/nuclei from this analysis are from the three studies that used high throughput collection and barcoding techniques (the Sathyamurthy, Rosenberg, and Zeisel datasets). A comparison across studies revealed that these high throughput studies detected fewer genes per cell/nucleus than studies that used single well technical approaches (the Hayashi, Haring, and Baek datasets), and studies that used cells (the Hayashi, Haring, Zeisel, and Baek datasets) detected more genes per cell/nucleus but had higher levels of immediate early gene and stress gene expression than did studies that used nuclei (the Sathyamurthy and Rosenberg datasets) (Supplementary Fig. 2). These trends across technical approaches were expected based on other reports (reviewed^22^). Thus, integration has the potential to facilitate merged analysis and comparison amongst independent datasets by reducing (but not eliminating) the effects of technical differences between the studies.

To test whether the particular integration method may bias downstream results and alter cell type assessments, we also performed integration using three independent methodologies^24^: Harmony^25^, Conos^26^, and LIGER^27^ (Supplementary Fig. 3). In each case, highly reliable cell type results were observed, based on visual inspection of UMAP distributions and low Local Inverse Simpson Index (LISI) values for cell type coherence when compared to Seurat integration. Together, these analyses demonstrated that integration of publicly available datasets can be used to harmonize spinal cord sequencing data, preserve important biological differences between studies, and uncover a robust set of shared cell types.

### A harmonized atlas of major cell types

Next, we performed coarse clustering to define the major cell types of the mouse spinal cord (Fig. 1d, e and Supplementary Fig. 2). Sixteen major types were identified that represent all known classes of spinal cord cell types. These cell types are: (1) oligodendrocyte precursor cells; (2–3) two maturational stages of oligodendrocyte progenitors; (4–5) two types of oligodendrocytes that likely correspond to myelinating and mature cell types and that blend into each other; (6) Schwann cells; (7) peripheral glia; (8–9) two types of meninges that likely correspond to vascular leptomeningeal cells and arachnoid barrier cells; (10) ependymal cells that surround the central canal; (11–12) two types of astrocytes that likely correspond to a major population of regular astrocytes and a minor population of Gfap-expressing proliferating/activated/white matter astrocytes; (13–14) two types of vascular cells that likely correspond to endothelial cells and pericytes; (15) microglia; and (16) neurons, which are discussed in detail below.

As expected, the cell types that were derived from each study corresponded to the techniques used to isolate the cells or nuclei (Supplementary Fig. 2e). The three studies that FACS sorted neurons from the spinal cord (Hayashi, Haring, and Baek datasets) predominantly gave rise to cells in the neuronal sub-clusters, as well as non-neural cells that most likely represented doublets. Moreover, among the three studies that examined all cell types, the early postnatal Rosenberg study showed enrichment of immature cells of oligodendrocyte lineage relative to the adult Sathyamurthy study, while the adolescent Zeisel study showed an intermediate distribution. The only study to dissect the spinal cord including the dorsal and ventral spinal roots (the Sathyamurthy dataset) was the only source of Schwann and peripheral glia cells that would be located in these roots.

### Overview of harmonized neuronal cell types

**Fig. 2:**
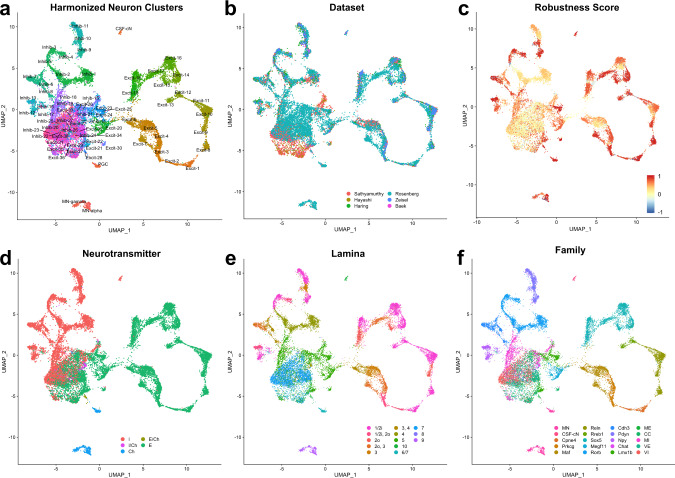
Harmonized atlas of 69 populations of spinal cord neurons. **a** UMAP presentation of 19,353 neuronal cells/nuclei of the postnatal mouse spinal cord, colored and annotated by cell type cluster. **b**–**f** The same cells/nuclei, colored by the study of origin (**b**), by robustness (silhouette) score (**c**), a neurotransmitter (**d**), lamina (**e**), and family (**f**). I inhibitory, I/Ch inhibitory cholinergic, Ch cholinergic, E/Ch excitatory cholinergic, E excitatory, MN motoneurons, ME mid excitatory, CC Clarke’s Column (*see main text for note on this designation), MI mid inhibitory, VE ventral excitatory, VI ventral inhibitory. Laminae were assigned based on in situ hybridization validation experiments and are colored by the approximate depth from the dorsal surface of the cord (hot pink to violet). See main text for description of neuronal families.

We next focused our analysis on neuronal populations to further probe their impressive diversity and to define a reference set of cell types for understanding the spinal cord cellular basis of behavior. Based on the coarse cell type assignments above, we selected and clustered all neuronal cells/nuclei (Supplementary Fig. 4). Preliminary analysis revealed that putative dorsal horn clusters separated well in principal component space while putative mid and ventral horn clusters did not, which prompted us to perform a targeted sub-clustering of all mid and ventral cells/nuclei (see Methods). In total, 69 neuronal clusters were identified (Fig. 2, Supplementary Figs. 4 and 5, Supplementary Table 2, and Supplementary Movie 1) and the neurotransmitter status and putative regional location (dorsal horn, mid-region, ventral horn) were determined by marker gene expression and comparison to the original six studies. Subsequent validation studies confirmed these determinations (see below). We observed 20 dorsal excitatory clusters, 14 dorsal inhibitory clusters, 10 deep dorsal/mid excitatory clusters, 7 deep dorsal/mid inhibitory clusters, 8 ventral excitatory clusters, 6 ventral inhibitory clusters, 3 cholinergic motoneuron clusters, and 1 cluster of the cerebrospinal fluid contacting neurons (CSF-cN).

To determine the robustness of these clusters, we used a bootstrapped co-clustering test of the consistency with which cells and nuclei in each cluster remain together upon repeated clustering (Fig. 2c and Supplementary Fig. 5). Dorsal clusters showed very high robustness with this measure, whereas mid and ventral clusters showed low to moderate robustness. This general feature was consistent with previous observations and likely reflects similar patterns of gene expression amongst mid and ventral clusters^1,4^.

To assess how these neuronal clusters relate to previously characterized transcriptomic spinal cord cell types, we first focused on the original clusters from the Sathyamurthy and Haring datasets because these two studies included a common set of cell types (dorsal horn neurons) and provided the most analysis, annotation, and marker validation for their respective cell types. Some ventral neurons from the Sathyamurthy dataset appeared in low-quality clusters that were discarded from the harmonized analysis due to low counts of genes per cell/nucleus and a lack of marker genes, whereas some neurons from the Haring dataset were classified as non-neural cell types or appeared in doublet clusters that were also discarded from the harmonized analysis. Nevertheless, we found that cells/nuclei from the original studies were distributed into the harmonized clusters in coherent patterns that facilitated the registration of the original clusters based on their distance in the neuron principal component space (Supplementary Fig. 6). We next compared the harmonized clusters to the clusters reported in a recent study by Blum et al. which focused on spinal motoneurons but also included many interneurons and glia. We found general agreement between the clusters that they reported and our harmonized analysis with the following differences (Supplementary Fig. 7). First, they described many more sub-types of motoneurons, similar to the work of Alkaslasi et al., and we incorporated both of these studies and performed an expanded analysis of motoneuron sub-types (see below). Second, most excitatory or inhibitory clusters that Blum et al. described corresponded to multiple refined harmonized clusters. Third, Blum et al. cluster “0” was described as inhibitory neurons but likely included both inhibitory and excitatory ventral neurons. And fourth, there were a few putative mis-annotation errors in the Blum et al. dataset: cluster “22” was not annotated but likely corresponded to ependymal cells; cluster “24” was annotated as inhibitory interneurons but likely corresponded to oligodendrocyte precursor cells; and cluster “35” was annotated as oligodendrocytes but likely corresponded to a mix (or doublets) of oligodendrocyte precursors/progenitors and other glial cell types such as astrocytes. Finally, we compared all marker genes that we highlight in this paper (those in Table 1 and all figures) to their patterns in a recent spatial transcriptomics analysis of the spinal cord^28^ and to the Allen^29^ and Gensat^30^ expression databases and found general concordance between these resources (Supplementary Table 4). Together, this analysis reveals the overall reproducibility of single-cell sequencing atlases of the spinal cord but also highlights the power of integrating many sources of information to obtain the most refined and robust cell types and the importance of having an annotated reference atlas to facilitate cell type analysis in future work.

### General trends in molecular identity relationships amongst neuronal populations

**Fig. 3:**
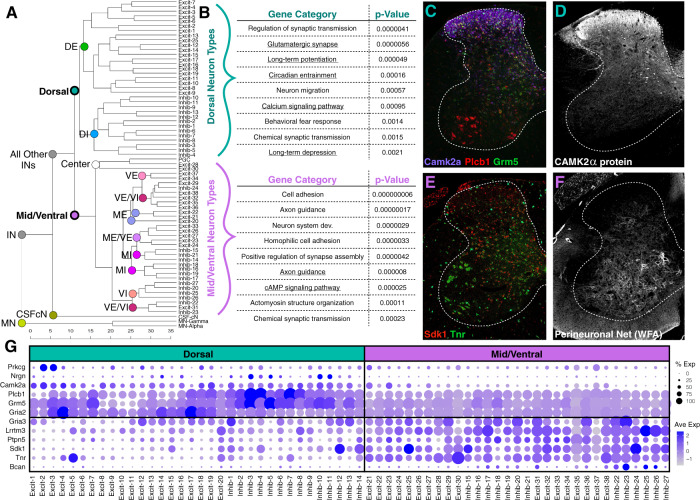
Trends in dorsal-ventral organization of spinal cord neuron types. **a** Dendrogram showing the relationships between the 69 neuronal cell types based on their distance from each other in the 50-dimensional principal component (PC) space. MN motoneuron, IN interneurons (and projection neurons), CSF-cN cerebrospinal fluid contacting neurons, DE dorsal excitatory, DI dorsal inhibitory ME mid excitatory, MI mid inhibitory, VE ventral excitatory, VI ventral inhibitory, center represents a group of 3 cell types located near lamina X–the center of the spinal cord. **b** Differential gene expression tests (ROC) were used to compare overall gene expression between the dorsal cell types and mid/ventral cell types and significant gene lists were analyzed by gene ontology (GO) term searches with GO DAVID using molecular function and biological process terms, as well as KEGG pathway lists (which are underlined) and the top terms for each cell class are shown. **c**–**f** Validation of differentially expressed genes using RNA in situ hybridization (**c**, **e**), antibody staining (**d**), or WFA-lectin staining (**f**). 20x tiled images, with brightness and contrast adjusted. All images are representative of the pattern observed in at least 3 sections each from *N* = 3 animals. Scale bars are 200 μm. **g** Dot plots showing expression of plasticity-related genes in each harmonized cluster, in which dot color intensity corresponds to average expression level (Ave Exp) and dot size corresponds to the percent of each cluster that expressed the gene (% Exp).

We next sought to examine the major features that govern the broad molecular identity relationships amongst spinal cord neurons. We used a dendrogram analysis of the distance between the clusters within the 50-dimensional principal component space. This revealed that the primary distinctions within spinal interneurons/projection neurons (non-motoneurons) were based on spatial location in the dorsal horn or mid/ventral regions of the spinal cord. Interestingly, this bifurcation occurred even before cell types split by neurotransmitter status (for example into dorsal excitatory or dorsal inhibitory types), a core feature of neuronal identity. This analysis also revealed that putative dorsal clusters were well separated from each other by long dendrogram branches, while putative mid and ventral clusters were much closer to each other in this reduced gene expression space (Fig. 3a). Intriguingly, neurons that are located at the spatial mid-point between the dorsal and ventral sides of the cord (preganglionic cells and two excitatory populations near the central canal) were organized as a single branch (Fig. 3a; center), further underscoring the importance of spatial distribution as an organizing principle in the spinal cord.

We next performed differential expression between dorsal and mid/ventral neuron types and compared the signature gene expression profiles by gene ontology analysis to uncover the broad molecular differences that distinguish these classes. Remarkably, we found that genes related to plasticity were significantly enriched in the dorsal horn of the spinal cord. This included (1) genes that were widely expressed in the dorsal horn but not in the ventral horn, such as Camk2a which has well-established roles in long-term potentiation (LTP)^31^; (2) genes that were present at higher levels in the dorsal horn than the mid/ventral horn, such as Grm5 which encodes the mGluR5 receptor which has been linked to meta-plasticity^32^, Plcb1 (PLCβ), and Gria3 (GluR3); and (3) genes with restricted expression in particular dorsal horn neuron types such as Prkcg (PKCγ), Kcnip3 (DREAM), Nrgn, and Nos1 (nNOS) (Fig. 3b, c, d, g). In contrast, genes related to structural adhesion and stability were enriched in the ventral horn of the spinal cord, including (1) genes related to cell-cell adhesion such as Lrrtm3, Cntn5, Cdh18, and Sdk1; (2) genes related to perineuronal net components such as Bcan (Brevican) and Tnr (Tenascin R), and genes related to limiting the signal transduction pathways associated with LTP such as Ptpn5 (STEP)^33^ (Fig. 3b, e, f, g). We validated the differentially expressed patterns of several of these genes using in situ hybridization and also at the protein level for CAMK2α and WFA-lectin to reveal perineuronal nets, thereby confirming predicted gene expression signatures that would differentially regulate plasticity in the dorsal and ventral horns of the spinal cord (Fig. 3c–f). Thus, we discovered general differences in the relationships between clusters in the dorsal versus the ventral horn and molecular trends that could confer differential plasticity control in these two regions.

We also performed a similar analysis to compare gene expression between excitatory and inhibitory classes of spinal neurons. As expected, genes involved in neurotransmitter status were detected (such as Pax2 and Gad2) but we also observed consistent differential expression between excitatory and inhibitory neurons for a pair of calcium channels (Cacna2d3 and Cacna2d1) and a pair of synaptic adhesion molecules that promote repulsion to limit homophilic interactions (Dscam and Dscaml1) (Supplementary Fig. 8).

### Detailed cluster analysis and marker validation for harmonized neuronal cell types

**Fig. 4:**
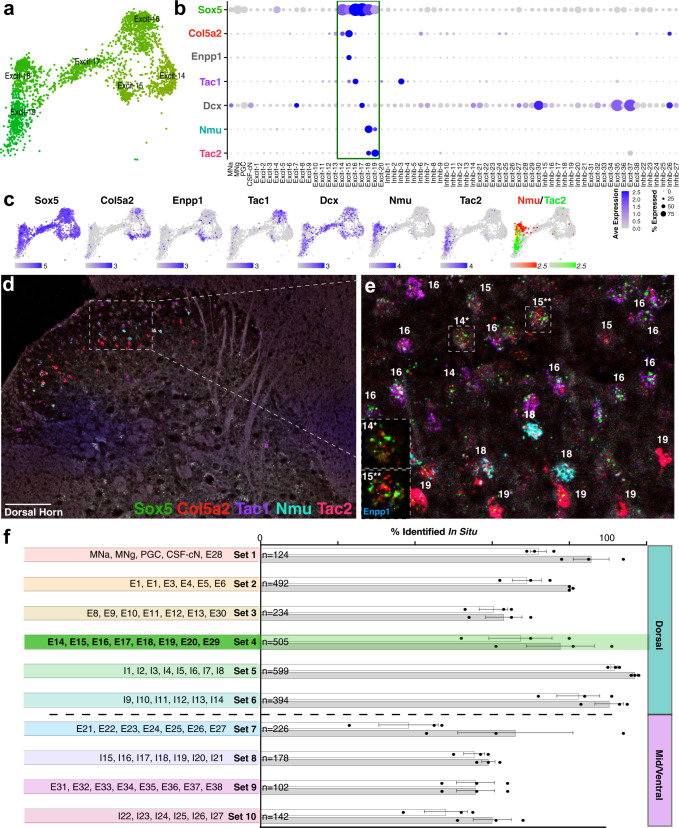
Family structure and in situ validation for adult spinal cord tissue. **a** UMAP for neuronal cell types Excit-14 through Excit-19. **b** Dot plot of the distribution of selected marker genes across the 69 neuronal clusters in which dot color intensity corresponds to average expression level (Ave Expression) and dot size corresponds to the percent of each cluster that expressed the gene (% Expressed). **c** Feature plots of each gene expression pattern in Excit-14 through Excit-19. Expression levels are indicated by color intensity, with the maximum level indicated below each plot. The co-expression of Nmu (red) and Tac2 (green) are shown in the right-most plot, with expression levels cut-off at a maximum of 2.5 to highlight co-expressing cells in yellow. **d**, **e** RNA in situ hybridization of selected marker genes Sox5, Col5a2, Tac1, Nmu, Tac2 on an adult mouse lumbar spinal cord section. Cells were assigned to individual excitatory clusters with cluster number identity shown based on marker gene expression. Inset show representative cells of Excit-14 (14*) and Excit-15 (15**) with in situ hybridization for Sox5 (green), Col5a2 (red), Enpp1 (blue). 20x tiled images, with brightness and contrast adjusted. All images are representative of the pattern observed in at least 3 sections each from *N* = 3 animals. Scale bar is 100 μm in (**d**) and 25 μm in (**e**). **f** Quantification of the cells in adult spinal cord tissue that could be defined using sets of marker genes in situ. The cell types analyzed by each set of genes are shown on the left, the number of cells counted for each set are shown at the base of the bars, and the percent of counted cells are shown for each animal (*N* = 3, replicates and mean ± standard error) that could be confidently assigned to a single cluster (white bars), or that could be assigned to a single cluster or to pair of closely related clusters (gray). For each set, the coarse criteria for counting total cells are specified in the Methods. Set 4, which includes the Sox5 family clusters, is highlighted in green as an example.

Next, we sought to characterize the individual clusters at a molecular level and to define their marker genes. There are multiple approaches for identifying cell type markers based on single-cell data. Commonly used methods such as the Wilcox Rank Sum test and Area Under the Curve Receiver Operating Characteristic (AUROC) analysis use differential expression to identify genes that are enriched within one identified cell cluster as compared to all other clusters and we used this approach to generate candidate markers for each cluster (Supplementary Table 1). However, these approaches do not prioritize markers that are shared between related clusters or those markers that are well-established for a given tissue, nor do they produce an efficient final set of markers that can be used to define neuronal cell types for use in other types of experiments. To overcome these obstacles, we therefore used a combination of Wilcox and ROC individual cluster markers, Wilcox and ROC markers for dendrogram branches, and established markers from the literature to generate a panel of combinatorial markers for spinal cord neurons that follows a family name and given name analogy. For example, Excit-14 through Excit-19 comprise the Sox5 family. They are distinguished by expression of Col5a2 (Excit-14), Col5a2 and Enpp1 (Excit-15), Col5a2, Enpp1, and Tac1 (Excit-16), Dcx expression and being present almost exclusively at early post-natal stages (Excit-17), Nmu (Excit-18), and Tac2 (Excit-19) (Fig. 4A, B).

To determine whether this panel of markers corresponded to in situ gene expression patterns and to define the anatomical distribution of each cluster, we performed high-content in situ hybridization with combinatorial sets of marker gene probes including both known and not previously described marker genes (Supplementary Table 3). While the harmonized analysis above included a range of tissue ages, all validation work was done in the adult lumbar spinal cord to test whether predicted gene expression patterns are accurate and whether they can be used in the adult context to study cells involved in the mature function. We tested 95 unique genes and analyzed gene expression in ten overlapping sets of 12 genes each. For each set, hundreds of cells were counted from three spinal cords and their locations mapped by lamina with examples shown in Fig. 4 and Supplementary Fig. 9. (Details for the counting procedure including are described in the “Methods”.)

Using this approach, 79 genes (out of 95) showed reliable expression in the adult spinal cord (Supplementary Table 3) and 71% of neurons in the adult lumbar spinal cord could be identified as belonging to one of the 69 neuronal clusters (2057/2894 total). An additional 9% of neurons could be identified as belonging to pairs of closely related clusters (266/2894 total). Of note, the ability to use defined markers to identify cell types in tissue varied by combinatorial gene set such that dorsal sets could be more readily assigned based on in situ gene expression than ventral sets (Fig. 4F). This suggests that the distinction between dorsal and ventral neurons that we described above is not limited to the sequencing data but exists in the adult spinal cord tissue. This detailed in situ hybridization analysis also revealed the in-tissue prevalence and laminar location of each of the lumbar adult neuronal cell types (Table 1) and can serve to translate single-cell sequencing data back into tissue-based analysis.

**Table 1:** Cell-type census of 69 populations of spinal cord neurons.

Cluster	Lamina	%	NT	Family	Individual Markers			Putative Lineage
**MN-alpha**	9	1.1	Chat	MN	Spp1	Poln	Tns1	MN
**MN-gamma**	9	0.5	Chat	MN	Esrrg	Htr1f	Tns1	MN
**PGC**	7-IML		Chat	MN	Gfra3	Nos1	Fbn2	MN
**CSF-cN**	10	0.3	Slc6a1	CSF-cN	Pkd2l1			V2b.2/V2b.4
**Excit-1**	1/2o	0.6	Slc17a6	Cpne4	Dach2	(Cck)		dI3.3/dI5
**Excit-2**	1/2o/2i	3.7	Slc17a6	Cpne4	Prkcg	(Rorb)		dI5.4
**Excit-3**	1/2o/2i	3.8	Slc17a6	Prkcg	(Cck)	Calb1		dI5.4
**Excit-4**	2i/3	2.8	Slc17a6	(Prkcg)	Nts	Calb1-hi		dI5.4
**Excit-5**	2i/3/4	3.2	Slc17a6	Maf	Cck			dI5.4
**Excit-6**	3/4	2.4	Slc17a6	Maf	Rorb	Cpne4		dI5.4/dI5.5
**Excit-7**	N/A	N/A	Slc17a6	Maf	Dcx	(vGlut3)		dI5.4/dI5.5
**Excit-8**	1/2	1.4	Slc17a6	Reln	Trhr	(Car12)	(Grp)	dI3.3/dI5.4
**Excit-9**	1/2/3	1.7	Slc17a6	(Reln)	Grp	Calb2		dI5.4/dI5.5
**Excit-10**	1/2	2	Slc17a6	Reln	Car12	Nmur2		dI5.4
**Excit-11**	N/A	0	Slc17a6	Reln	Car12	Gabra2		dI5.4
**Excit-12**	1/2	0.2	Slc17a6	Rreb1	Satb1	Zim1		dI5.5
**Excit-13**	2i/3	0.7	Slc17a6	Rreb1	Nmur2	(Satb1)		dI5.5
**Excit-14**	1/2o	1.7	Slc17a6	Sox5	Col5a2			(dI5)
**Excit-15**	1/2/3	0.2	Slc17a6	Sox5	Col5a2	Enpp1		(dI5)
**Excit-16**	1/2o (2i-4)	6.5	Slc17a6	Sox5	Col5a2	Enpp1	Tac1	(dI5)
**Excit-17**	N/A	N/A	Slc17a6	Sox5	Dcx			(dI5)
**Excit-18**	1/2o (2i-4)	2.7	Slc17a6	Sox5	Nmu	(Tac2)		(dI5)
**Excit-19**	2i (3/4)	1.9	Slc17a6	Sox5	Tac2	(Nmu)		dI5.4/dI5.5
**Excit-20**	4/5	2	Slc17a6	Megf11	Mdga1			dI2.1/dI5.5
**Inhib-1**	3 (1-4)	7.4	Slc6a1	Rorb	Sorcs3	(Nppc)	(Runx2)	dI4.3
**Inhib-2**	3 (1-4)	10.3	Slc6a1	(Rorb)	Adamts5	Klhl14	Sorcs3	dI4.3
**Inhib-3**	1-4	3	Slc6a1	Rorb	Nppc	Nrgn		dI4.3
**Inhib-4**	1/2o/2i	0.4	Slc6a1	Rorb	Rxfp2			dI4.3
**Inhib-5**	1/2o (3)	1	Slc6a1	Rorb				dI4.3
**Inhib-6**	3/4 (1/2o)	1.3	Slc6a1	Cdh3				dI4.4
**Inhib-7**	2i/3 (1-4)	3.6	Slc6a1	Cdh3	Kcnip2	Pvalb		dI4.4
**Inhib-8**	3/4	0.5	Slc6a1	(Cdh3)	Klhl14-hi			dI4.4
**Inhib-9**	1/2o (2i/3)	1.6	Slc6a1	Pdyn	(Rorb)	(Rspo3)		dI4.1/dI4.4
**Inhib-10**	3 (1-5)	9.7	Slc6a1	Pdyn	Gal	Mlxipl	Rspo3	dI4.1/dI4.4
**Inhib-11**	1/2o/2i/3	0.9	Slc6a1	Pdyn	Gal	(Rorb)	Nrgn	dI4.1/dI4.4
**Inhib-12**	1/2o/4	1.8	Slc6a1	Npy	(Vgf)			dI4.6
**Inhib-13**	1/2o/2i	2.1	Slc6a1	Npy	Qrfpr			dI4.6
**Inhib-14**	4	0.1	Slc6a1	Chat	Slc6a5	Nos1		dI4.1/dI4.6
**Excit-21**	4/lat 5	0.5	Slc17a6	ME/Lmx1b	Zfhx3	Nms		dI5.5
**Excit-22**	4/5/6	0.1	Slc17a6	ME/Lmx1b	Zfhx3			dI3.3/dI5.5
**Excit-23**	4/med 5	1.2	Slc17a6	ME/Lmx1b	Nfib	Cep112		(dI5)
**Excit-24**	4/5/6	0.7	Slc17a6	ME/Lmx1b	(Nfib)	(Cep112)		(dI5)
**Excit-25**	4/5/6	0	Slc17a6	ME/Lmx1b	Nfib	Prox1		(dI5)
**Excit-26**	4	0.1	Slc17a6	ME	Nfib	(Prox1)	(Satb1)	(dI1/dI2)
**Excit-27**	4/5	1.3	Slc17a6	ME	Adamts2	Cep112)		(dI2)
**Excit-28**	10	0.1	Chat	ME	Pitx2	Pou6f2	Onecut2	V0*
**Excit-29**	5/6	0.3	Slc17a6	ME	Onecut2	Pmfbp1		(V0)
**Excit-30**	5	0.8	Slc17a6	CC#	Gbx2	Neurod2+	Pou6f2	V2a.1
**Inhib-15**	med 5	1.1	Slc6a5	MI	Prox1	Gabra1	Nfib	V1.7
**Inhib-16**	med 5	0.6	Slc6a5	MI	Gpc3	(Rorb)	Sema5b	dI4.6
**Inhib-17**	N/A	N/A	Slc6a5	MI	Satb2			dI4.4/dI4.6/(dI6)
**Inhib-18**	5/6	0.5	Slc6a5	MI	Sema5b			dI4
**Inhib-19**	med 5	0.5	Slc6a5	MI	Ccbe1	Pou6f2		dI4.4
**Inhib-20**	5/6	1	Slc6a5	MI	Tfap2b			dI4.6/V1.1/(dI6)
**Inhib-21**	4/med 5	0.8	Gad2	MI	Nfib	Pax6		dI4.6/V1.6/(dI6)
**Excit-31**	6/7/8	0.3	Slc17a6	VE	Lhx9	Gm26673	Syt2	(dI1/dI2)
**Excit-32**	6/7/8	0.4	Slc17a6	VE	Lhx9	Prlr	Mdga1	dI1/dI2/dI3
**Excit-33**	N/A	N/A	Slc17a6	VE	Lhx9			dI2.1
**Excit-34**	6/7/8	0.4	Slc17a6	VE	Bnc2	Pou6f2	Lhx2	dI1/dI2
**Excit-35**	6/7	0.5	Slc17a6	VE	Vsx2	Pou6f2	Vamp1	V2a*
**Excit-36**	6/7	0.3	Slc17a6	VE	Vsx2	Esrrg	(Gm26673)	dI1/dI2/V2a
**Excit-37**	7	0.8	Slc17a6	VE	Vsx2	Shox2*		V2a*
**Excit-38**	N/A	N/A	Slc17a6	VE	Sim1	Rnf220		V3*
**Inhib-22**	7	0.1	Slc6a5	VI	Foxp2	(Esrrb)		(dI6)/V1.3
**Inhib-23**	7/8	0.6	Slc6a5	VI	Foxp2	Esrrbb+	Gm26673	(dI6)/V1.3
**Inhib-24**	7	0.6	Slc6a5	VI	Pou6f2	Nr5a2		V1
**Inhib-25**	7/8	1.1	Slc6a5	VI	Esrrb	(Pvalb)		(dI6)/V1
**Inhib-26**	ventral 7	0.5	Slc6a5	VI	Chrna7	Calb1	(Pvalb)	V1.1/V1.2
**Inhib-27**	7	0.3	Slc6a5	VI	Foxp2	(Gata3)	Pax2-hi	(dI6)/V1/V2b

The lamina, prevalence, neurotransmitter marker gene, family, individual marker genes, and putative embryonic lineage for each neuronal cluster are shown. The clusters are color-coded to correspond approximately to their color in Fig. 2a. The prevalence of each cluster was determined by counting the confidently assigned cells of each type based on RNA in situ hybridization on sections from three animals and are presented as the percent of the total number of confidently assigned neurons. Genes in parenthesis are expressed at lower levels. Genes in gray were not validated (due to probe failure, being present only in postnatal animals, or were not included in the analysis). + denotes relatively higher expression. # denotes a possible identity of Clarke’s column (CC). * denotes a marker that was validated using RNAScope V2 but did not work in the RNAScope Hiplex assay.

The cell type markers, typical laminar distribution, prevalence, and putative embryonic lineage (described below) of each cluster are shown in Table 1, Fig. 4, and Supplementary Fig. 9.

The motoneuron (MN) family includes alpha motoneurons (MNa) which had relatively higher levels of Poln and Spp1^34^, gamma motoneurons (MNg) which had relatively higher levels of Esrrg and Htr1f^35^, and the related preganglionic cells (PGC) which expressed Gfra3, Nos1, and Fbn2^36,37^. This family was only comprised of nuclei from the Sathyamurthy and Rosenberg datasets but we could not detected refined sub-populations of motoneurons. However, this included only 565 MNs. Recently, data from Blum et al.^10^ and Alkaslasi et al.^11^, focusing only on cholinergic neurons, became available. Therefore, we incorporated this data and performed a targeted analysis of a merged set of 23,032 spinal motoneurons. This larger dataset, combined with the barcoding of the Alkaslasi study enabled the identification of distinctly localized MN subtypes that were not previously resolved. PGCs were clustered into 23 subtypes that varied by spinal cord level and MNa were clustered into 14 subtypes that also varied by spinal level. For example, we found that digit-innervating motoneurons, expressing Cpne4 and Fign^38^, separated into two subtypes, one found in limb-innervating regions (both cervical and lumbar) and one that was specifically localized to the lumbar spinal cord^39,40^ (Supplementary Fig. 9A and Supplementary Fig. 10).

Cerebrospinal fluid contacting neurons (CSF-cN) were distinguished by Pkd2l1 and Pkd1l2. This population has been suggested to be involved in postural control in zebrafish^41–45^. This cluster was very distinct from other neuronal populations, inhibitory, and also expressed the early neuron marker Sox2 and the V2b lineage markers Gata2 and Gata3, suggesting an immature phenotype. (Supplementary Fig. 9A).

The dorsal excitatory cell types were comprised of the following families:

The Cpne4 dorsal, excitatory family was comprised of Excit-1 and Excit-2. Excit-1 was a rare subset, both in the harmonized clusters and in the in situ counts, that also expressed Dach2. Excit-2 was more prevalent and co-expressed Prkcg as well as Cbln2. This family had markers of interneurons suggested to be involved in mechanical itch^46,47^. (Supplementary Fig. 9B).

The Prkcg dorsal, excitatory family was comprised of Excit-3 and Excit-4 and likely corresponded to neurons involved in light static touch and allodynic pain in pathological situations^46,48–56^. Prkcg is a classic marker gene in the spinal cord and defined this family together with the neuropeptides Cck and Trh (Excit-3) and Nts (Excit-4). Both subsets also expressed Calb1, although it was not specific to these clusters. This family was also close to Excit-7, an immature cluster grouped with the Maf family. Of note, there were two discrepancies in this family between the sequencing data and the in situ hybridization data: Cck was present at high levels in the Excit-3 in the sequencing data but we did not detect Cck in most Prkcg-expressing cells of the adult spinal cord and Prkcg was not enriched in Excit-4 in the sequencing data but was readily detected with Nts and Calb1 in this cluster in adult tissue. (Supplementary Fig. 9B).

The Maf dorsal, the excitatory family was comprised of Excit-5, Excit-6, and Excit-7 which expressed markers of neurons involved in light touch^46,48,50,56,57^. All three clusters expressed enriched levels of Maf and Rora (which was broadly expressed in many other clusters at lower levels). Excit-5 also expressed Pvalb and Cck, Excit-6 expressed Rorb and Cpne4, and Excit-7 was distinguished by having only nuclei from the Rosenberg dataset and expressed the immature neuron marker Dcx, suggesting an immature phenotype. The similarity of Excit-7 with Excit-3, Excit-4, Excit-5, and Excit-6 suggests a shared lineage relationship between these families. This family also expressed low levels of Slc17a8 (vGlut3). (Supplementary Fig. 9B).

The Reln dorsal, excitatory family was comprised of Excit-8, Excit-9, Excit-10, and Excit-11 and expressed markers indicative of a role in chemical itch sensation^58–62^. These clusters expressed enriched levels of Car12 (in particular in Excit-9 and Excit-10), the neuropeptide receptors Trhr (Excit-8), Npr1 (Excit-9 and Excit-10), and Nmur2 (Excit-10), and the neuropeptide Grp (Excit-9). (Supplementary Fig. 9C).

The Rreb1 dorsal, excitatory family was comprised of Excit-12 and Excit-13. These clusters also expressed Satb1 and either Zim1 (Excit-12) or Nmur2 and Crh (Excit-13). (Supplementary Fig. 9C).

The Sox5 dorsal, the excitatory family was comprised of Excit-14, Excit-15, Excit-16, Excit-17, Excit-18, and Excit-19, and expressed markers suggestive of a role in coping pain and mechanical nociception^46,52,56,63–67^. Within this family, Excit-14 and Excit-15 were slightly separated and also similar to the Rreb1 family clusters and expressed Col5a2 (Excit-14) or Col5a2 and Enpp1 (Excit-15). Excit-16, Excit-18, and Excit-19 expressed the neuropeptides Tac1 (Excit-16), Nmu-hi/Tac2-lo (Excit-18), and Tac2hi/Nmu-lo (Excit-19). Excit-17 included almost exclusively nuclei from the Rosenberg dataset and showed enriched expression of the immature neuron marker Dcx. (Fig. 4).

The Megf11 cluster (Excit-20) displayed features of dorsal excitatory neurons and mid excitatory neurons, being located in lamina 4/5 and being grouped with mid neurons in principal component space in the UMAP and dendrogram analysis. It expressed Megf11 and Mdga1.

The dorsal inhibitory cell types were comprised of the following families:

The Rorb and Adamts5 dorsal, the inhibitory family was comprised of Inhib-1, Inhib-2, Inhib-3, Inhib-4, and Inhib-5, with markers of neurons involved in the dampening of dynamic touch^48,68,69^. Each of these clusters, except Inhib-2, expressed Rorb. Inhib-2 is grouped with this family based on its proximity in principal component space, as reflected in the UMAP and dendrogram analysis. In addition to Rorb, Inhib-1 expressed Sorcs3, Inhib-3 expressed Nppc as well as Nrgn, Inhib-4 expressed Rxfp2, and Inhib-5 did not express these other genes. Inhib-2 expressed Sorcs3 and Adamts5. (Supplementary Fig. 9D).

The Cdh3 dorsal, inhibitory family was comprised of Inhib-6, Inhib-7, and Inhib-8 and are likely to be involved in the dampening of dynamic touch and therefore in mechanical allodynia^70–72^. Inhib-6 and Inhib-7 expressed Cdh3 and were distinguished by co-expression of Kcnip2 and Pvalb in Inhib-7. While Inhib-8 contained only low levels of Cdh3 in this analysis, Cdh3 expression was confirmed by in situ hybridization and this cluster was included in this family based on proximity in principal component space as reflected in the UMAP and dendrogram analysis. Inhib-8 also expressed Klhl14. (Supplementary Fig. 9D).

The Pdyn dorsal, inhibitory family was comprised of Inhib-9, Inhib-10, and Inhib-11 and expressed markers suggestive of a role in chemical itch^52,61,73–77^. Each of these clusters expressed Pdyn, while Inhib-10 also expressed Gal and Mlxipl and Inhib-11 also expressed Gal only. Of note, the clusters in this family also expressed Rorb and Nrgn. (Supplementary Fig. 9E).

The Npy dorsal, the inhibitory family was comprised of Inhib-12 and Inhib-13. Studies suggest this family’s markers identify neurons involved in mechanical itch and pain^73,78,79^. These clusters expressed Npy and were distinguished by low levels of Vgf (Inhib-12) or by expression of Qrfpr (Inhib-13). (Supplementary Fig. 9E).

The Chat dorsal inhibitor cluster Inhib-14 was a deep dorsal (lamina 4), inhibitory and cholinergic population and also expressed Nos1^80–82^.

The cell types of the mid-region of the spinal cord, the deep dorsal horn, were comprised of the following families and the clusters were generally less robust than dorsal clusters (Fig. 2b and Supplementary Fig. 5).

The mid excitatory (ME)/Lmx1b family was comprised of Excit-21, Excit-22, Excit-23, Excit-24, and Excit-25 and corresponds to cells suggested to be involved in pain^3,83^. These clusters expressed Lmx1b, suggesting a dI5/dIL^B^ embryonic origin. All of the clusters except Excit-25 expressed Tacr1 and Excit-21 also expressed Lypd1, suggesting that these are candidate ascending populations^3^. These clusters could also be distinguished by expression of Zfhx3 (Excit-21 and Excit-22) or Nfib (Excit-23, Excit-24, and Excit-25), which corresponded to lateral Zfhx3 and medial Nfib sub-types. Other markers sub-divided the clusters in a combinatorial manner, including Nms (Excit-21), Bcl11a (Excit-22 through Excit-25), Satb1 and Cdh23 (Excit-23, Excit-24, and Excit-25), Cep112 (Excit-23 and Excit-24), and Prox1 (Excit-25). Of note, nearly all of the cells and nuclei in this family were from the Rosenberg and Sathyamurthy datasets. (Supplementary Fig. 9F).

The remaining ME family was comprised of mid, excitatory clusters were comprised of Excit-26, Excit-27, Excit-28, and Excit-29. These clusters do not express Lmx1b, in contrast to the other mid excitatory family and are likely derived from dI1-3 or ventral embryonic lineages. Excit-26 expressed Nfib, Excit-27 expressed Adamts2, Excit-28 expressed Chat and Pitx2 and thus likely corresponds to V0c neurons, and Excit-29 expressed Pmfbp1. Excit-28 and Excit-29 also express Onecut2 and Pou6f2, potentially revealing a link with ventral cell types. Of note, nearly all of the cells and nuclei in this family were from the Rosenberg and Sathyamurthy datasets and Excit-26 in particular was predominantly from the Rosenberg dataset. (Supplementary Fig. 9A and F).

The Excit-30 cluster was marked by Gbx2, Neurod2, and Sp8 and there was partial evidence that it corresponded to Clarke’s column (CC). This cluster expressed multiple genes associated with Clarke’s column including Chmp2b, Syt4, Ebf3, Rgs4, and Enc1^6^. The Clarke’s column marker gene, Gdnf, was expressed at very low levels in the merged dataset, but was present in several Excit-30 cells. However, this cluster only contained two defined spinocerebellar cells from the Baek et al. dataset while the majority of this cluster was from the Hayashi dataset, arguing against Clarke’s column identity and also suggesting a V2 embryonic lineage. As the in situ hybridization experiments were performed on mid/lower lumbar spinal cord sections, we did not validate markers for this cluster.

The mid inhibitory cell types were grouped as one family comprised of Inhib-15, Inhib-16, Inhib-17, Inhib-18, Inhib-19, Inhib-20, and Inhib-21, which expressed the glycinergic marker Slc6a5 (with the exception of Inhib-21) and also the gaba-ergic marker Gad2, implicating these neurons in sensorimotor processing^84–86^. Inhib-15 expressed Prox1, Gabra1, and Nfib, Inhib-16 expressed Gpc3 and Sema5b, Inhib-17 expressed Satb2, Inhib-18 expressed Sema5b, Inhib-19 expressed Ccbe1 and Pou6f2, Inhib-20 expressed higher levels of Tfap2b as well as Zfhx3, and Inhib-21 expressed Nfib and was distinguished by having only Gad2 and not Slc6a5 and was mainly derived from the Rosenberg dataset. (Supplementary Fig. 9G).

In general, we found that the ventral clusters had less distinct gene expression patterns and were less robust than dorsal and mid clusters; therefore, the final identities of these clusters should be considered with caution. We identified several genes that contribute to overlapping gene expression patterns across clusters by being present in a spatial region of the cord and in diverse mid/ventral cell types. For example, Pou6f2 was expressed in the deep dorsal horn and in the dorsal part of the ventral horn and was enriched in mid-excitatory (Excit-21, Excit-28, and Excit 30), ventral excitatory (Excit-34 and Excit-35), and ventral inhibitory (Inhib-24) clusters that are located within this domain. Similarly, Nfib was expressed in the medial deep dorsal horn (mid) spinal cord and was enriched in both excitatory (Excit-23, Excit-25, and Excit-30) and inhibitory (Inhib-15 and Inhib-21) clusters. Of note, several cluster markers of ventral cell types, such as Sim1, were not observed in adult spinal cord tissue by in situ hybridization and while they are detected in the harmonized sequencing data, they likely represent lingering RNA from developmental samples.

The ventral, excitatory clusters were grouped as one family comprised of Excit-31, Excit-32, Excit-33, Excit-34, Excit-35, Excit-36, Excit-37, and Excit-38. Of these, Excit-31, Excit-32, Excit-33, and Excit-34 expressed low levels of Lhx2, Lhx9, and Isl1, potentially suggesting dorsal dI1/dI2/dI3 embryonic lineages for these clusters. These clusters could be distinguished by Gm26673, Syt2, and Prlr (Excit-31), Mdga1 and Prlr (Excit-32), and Bnc2 and Pou6f2 (Excit-34). Excit-33 was comprised almost entirely of nuclei from the Rosenberg dataset and may represent an immature cell type. Excit-35, Excit-36, and Excit-37 are likely derived from the V2a lineage, as they expressed Vsx2 (Chx10) and included many cells from the Hayashi dataset that sorted cells based on Chx10 genetic expression and thus may play a role in skilled reaching^87^. Excit-35 also expressed Vamp1, Pou3f1, Shox2, and Pou6f2 and Excit-36 expressed Esrrg. Intriguingly, many cells from the Baek dataset, which sorted cells based on spinocerebellar status were found in Excit-35, suggesting that this population includes ascending projection neurons that target the cerebellum. Excit-37 expressed low levels of the V3 marker gene Sim1 as well as Rnf220. (Supplementary Fig. 9H).

The ventral, inhibitory clusters were also grouped as one family that was comprised of Inhib-22, Inhib-23, Inhib-24, Inhib-25, Inhib-26, and Inhib-27. Each of these clusters expressed the glycinergic marker Slc76a5. Inhib-22 and Inhib-27 also expressed the gaba-ergic marker Gad2 as well as Pax2 and Pou6f2. They were distinguished by low levels of Gata3 expression in Inhib-27, which may represent V2b lineage. Inhib-23 and Inhib-25 expressed Foxp2 and Esrrb, suggesting they correspond to the Foxp2 clade of V1 lineage neurons described by Bikoff and colleagues^88^. They were identified by expression of Gm26673 and Pvalb in Inhib-23, which may suggest that this cluster included Ia-inhibitory neurons^89^. Inhib-24 expressed both Pou6f2 and Nr5a2, suggesting that this cluster corresponded to the Pou6f2/Nr5a2 clade of V1 lineage neurons^88^. Inhib-26 was the most robust ventral cluster and expressed the Renshaw marker genes Chrna2, Chrna7, and Calb1, suggesting that this cluster corresponded to Renshaw cells^89,90^. (Supplementary Fig. 9I).

### Developmental lineages of postnatal spinal neuron populations

Having established this harmonized atlas of postnatal through adult spinal cord neurons, we next asked if these cell types could be aligned with the cardinal classes of embryonic spinal progenitors. Developmental lineage has been a powerful and influential framework for categorizing spinal neurons, particularly within the ventral horn, and relies on the combinatorial expression code of transcription factors that specify distinct progenitor domains along the dorsal-ventral axis of the spinal cord. While recent work has revealed an impressive diversity of gene expression patterns within each cardinal class^2,88,89,91–97^, the contribution of these populations to adult neuronal classes and function is still not clear.

**Fig. 5:**
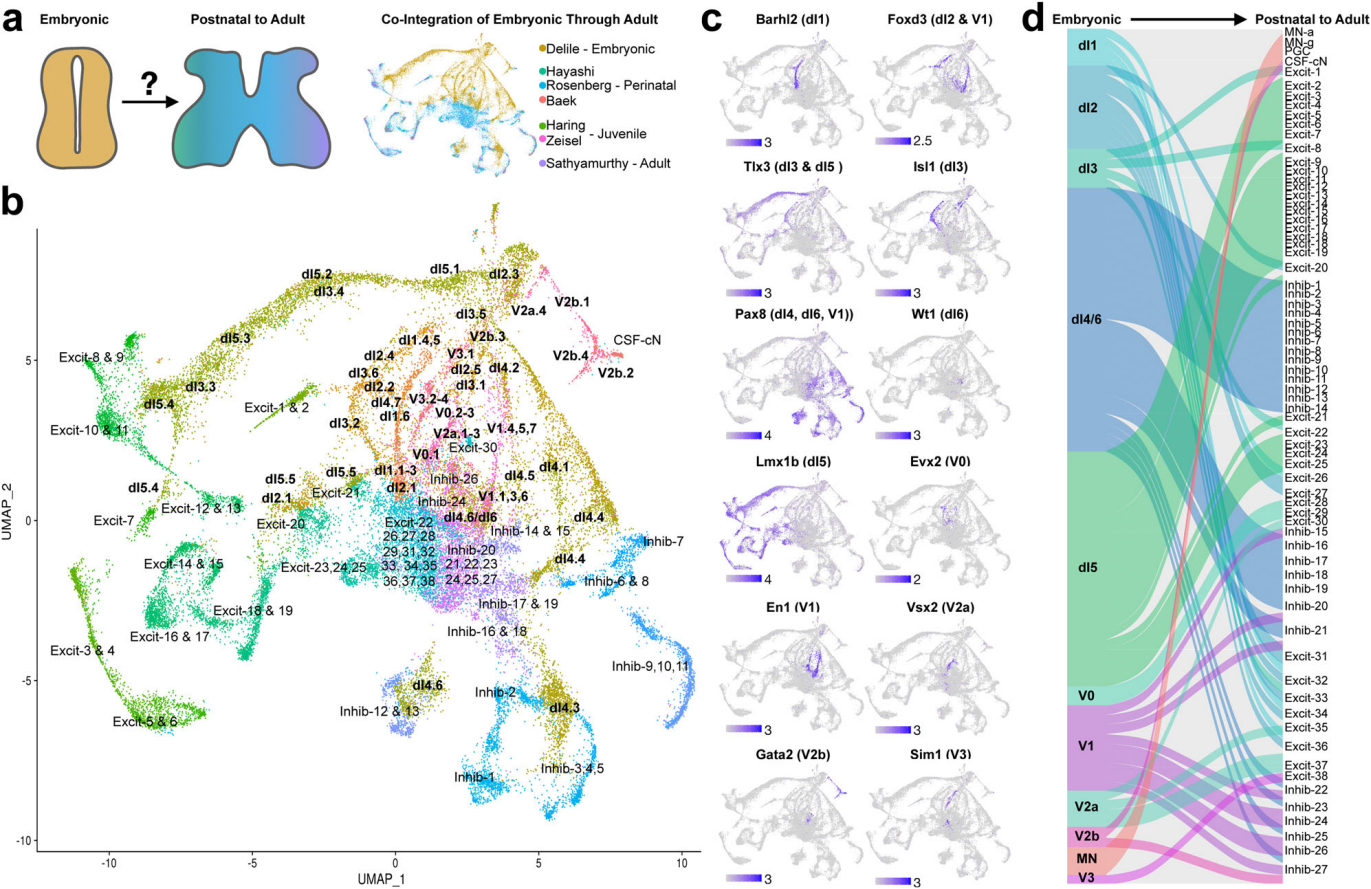
Co-integration of embryonic and postnatal through adult spinal cord neuronal types. **a** To reveal the temporal relationship between embryonic and postnatal through adult cell types, the Delile, and harmonized datasets were co-integrated and are shown in a UMAP, colored by dataset (right). **b** A UMAP of the co-integrated datasets, colored by clusters from the Delile et al. study (bold labels) or the harmonized analysis (regular font labels). Cluster annotations are repeated in cases in which a group of cells from a given cluster are located at a distance from the cluster centroid (ex. for dI4.6). **c** Feature plots of selected marker genes for the cardinal classes of spinal cord lineages. **d** Sankey plot of the relationships between embryonic lineages (left) and harmonized cell types (right) showing multiple examples of divergence and convergence.

We co-integrated the merged dataset of six postnatal studies with the neurons from a single cell sequencing atlas of e9.5 to e13.5 mouse spinal cord^7^ and analyzed these seven studies together (Fig. 5a). Dimensionality reduction with principal components (PC) was performed and the distances in PC-space between the centroid of each embryonic or postnatal cluster and every other cluster centroid was used to determine transcriptionally similar nearest neighbors between embryonic and postnatal cell types. In addition, the identity of the individual embryonic cells that were closest in PC-space to each cluster were also determined (Supplementary Table 6).

We found that long trajectories of embryonic cells led towards particular cluster families, which allowed us to infer developmental relationships (Fig. 5b). The molecular identities of the cardinal classes were confirmed by classic marker analysis (Fig. 5c) and these marker genes often extended into the postnatal cell type domains as well. A combination of centroid distance, nearest cells, and marker gene expression was used to assign an embryonic lineage for each harmonized neuron population (Table 1 and Fig. 5d).

The most striking finding from this analysis was the unexpectedly high degree of convergence of multiple lineages into postnatal cell types. Notable examples of this trend include: (1) Multiple dorsal excitatory cell types seemed to be derived from a mixed set of dI5 lineage cells (as expected) and an Isl1-negative, Tlx3-positive dI3.3/dI3.4 embryonic population (which was unexpected). Although Tlx3 is known to be a dorsal marker and a marker of dI3 neurons, the dI3 population is generally considered to give rise to an Isl1-positive population in the deep dorsal horn/intermediate zone^98–102^. (2) A subset of cells from the dorsal dI4.6/dI6 lineages contributed to most ventral inhibitory populations, which was unexpected though there have been reports of scattered dI4 Ptf1a-derived cells in the embryonic and perinatal ventral horn^103,104^, and the very small dI6 population is known to be ventrally located^105–107^. (3) Dual contributions of dI5.5 and dI2.1 to Excit-20 and of V1 and V2b to Inhib-27 were also observed. This analysis also supported a division of deep dorsal horn excitatory neurons into two overall groups (with the ME/Lmx1b family being more closely related to dI5, while the other ME clusters are more closely related to dI1-dI3) and a division of ventral excitatory neurons into two groups (with Excit-31 through Excit-34 being closer to dI1-3 and Excit-35 through Excit-37 being closer to V0, V2a, and V3 populations). Together, the joint analysis of spinal cord neurons from embryonic through adult stages began to align these two perspectives on cell types while also highlighting the complex relationships that exist between genetically defined cellular origins and mature transcriptional signatures.

### Using machine learning to classify spinal cord cell types

**Fig. 6:**
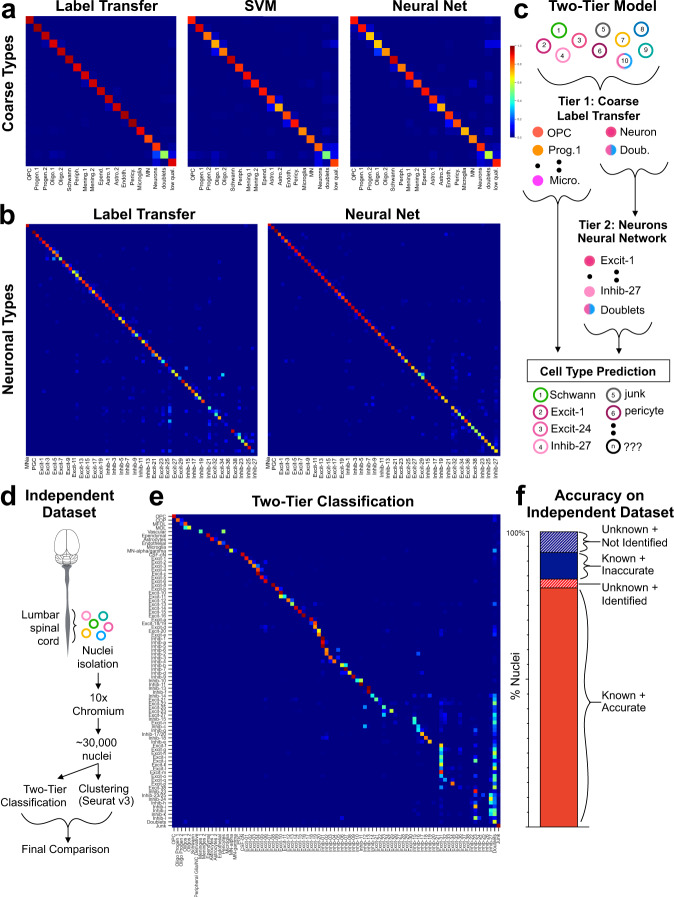
Computational classification of spinal cord cell types. **a** Confusion matrices of the F1 scores for the classification of coarse cell types using label transfer, a support vector machine (SVM), and a fully connected neural network (neural net), (blue = 0; maroon = 1). The actual cell types are in rows and the predicted cell types are in columns in the same order. **b** Confusion matrices of the F1 scores for the classification of fine neuronal sub-types using label transfer and a fully connected neural network. The actual cell types are in rows and the predicted cell types are in columns, both in the order presented in Table 1. Alternating cell types are labeled. **c** Model of the two-tiered classification approach in which all cells/nuclei are classified into coarse cell types using label transfer (also including low-quality junk and doublets). Subsequently, all cells/nuclei that were classified as neurons, motoneurons, or doublets by label transfer are further classified into 69 neuronal cell types (also including doublets). **d** Experimental design for generating an independent set of single nucleus RNA sequencing data. **e** Distribution plot showing how nuclei from each cluster (rows) were distributed into each of the harmonized cell types (columns), normalized by rows with dark blue = 0.0 fraction; maroon = 1.0 fraction). **f** Bar plot of the total counts of nuclei that were from known clusters and were correctly classified (81% of total), that were from known clusters and were incorrectly classified (9% of total), that were from unknown clusters but could be identified by their classification (3% of total), or that were from unknown clusters and could not be identified (7% of total). OPC oligodendrocyte precursor cell, progen.1 oligodendrocyte progenitor 1, progen.2 oligodendrocyte progenitor 2, Olig.1 oligodendrocyte 1, Olig.2 oligodendrocyte 2, Periph. peripheral glia, Mening.1 meninges 1, Mening.2 meninges 2, Epend. Ependymal cells, Astro.1 astrocytes 1, Astro.2 astrocytes 2, Endoth endothelial cells, Pericy pericytes, MN = motoneurons; low qual. low quality, MNa motoneurons alpha, PGC preganglionic cell.

We next sought a means to standardize and automate spinal cord cell type classification. First, we tested three strategies that have been used successfully to classify single-cell data from other tissues on their ability to classify spinal cord cells into coarse cell types. These were label transfer^23^, a support vector machine, and a fully connected neural network (with two hidden layers of 512 nodes and L2 regularization for each). It is important to note that each of these models were trained using cell type labels from the harmonized analysis because there is no existing gold standard for spinal cord cell identities. In this context, the first phase of the analysis that follows should be considered a feasibility study for machine learning classifiers on spinal cord single cell count data. The full merged dataset of 101,070 cells and nuclei was tested, including low-quality cells and nuclei and doublets, in order to represent the full range of input raw data. All three strategies performed well, with label transfer showing the best performance (overall accuracy of 89%), followed by the neural network (83%), and then the SVM (80%) (Fig. 6a and Supplementary Table 6).

Next, we tested label transfer and neural networks on a more refined and challenging task: the classification of 69 neuronal sub-types. For label transfer, two phases of analysis were performed (dorsal sub-types and then mid/ventral sub-types) because we found that this approach was important for clustering spinal cord neurons. For the neural networks, a non-exhaustive hand sweep of several hyperparameters was conducted, including network depth, optimizer, number of hidden nodes, and the number of training epochs, and seven different models were tested (see Methods and Supplementary Table 6). We found that a linear model (with no regularization and with an SGD optimizer) showed the best performance, with an overall test accuracy of 85% (Fig. 6b and Supplementary Table 6). The model showed very high confidence scores for correct predictions; however, performance varied with cell type prevalence suggesting a target for improving the model in the future (Supplementary Fig. 11).

How should the performance of this model be assessed and should we expect automated classification to achieve 100% accuracy? Perfect performance would require perfect and invariable biological data: discrete cell types that express completely distinct patterns of gene expression and experimental data without natural heterogeneity, doublets, low-quality cells, or other sources of indeterminate data. Knowing that this is not possible, we still sought to determine a benchmark performance guide for the classification of adult mouse spinal cord neurons using neural network models and considered four metrics of cluster definition and separation. We examined the relationship between the model performance for each cluster (F1 score) and (1) the co-clustering frequency of each cell type across 100 clustering iterations, (2) how distant each cluster was from its nearest neighbor in principal component space, and (3) the confidence with which clusters could be distinguished based on in situ marker expression (measured by in situ analysis sets of clusters) (Supplementary Fig. 12). We found that the model performance varied with the co-clustering frequency of each cluster and with the ability to identify cell types in situ and we propose that these measures can be used to set a reasonable expectation for neural network performance. Overall, neuronal cells/nuclei of a given type co-clustered together 65% of the time (average from Supplementary Fig. 5E) and a total of 70% of cells could be classified in situ (Fig. 4). In comparison, the model’s accuracy of 85% reveals the outstanding performance of this approach.

To develop a standardized pipeline for the classification of independent datasets unrelated to the original studies analyzed above, we considered a two-tiered approach that would take advantage of the strengths of both the label transfer for coarse classification (Tier 1) and a neural network model for classification of neuronal sub-types (Tier 2) (Fig. 6c). By combining these two methods, we improved overall performance by maximizing performance at both steps. We first selected all cells/nuclei that were assigned as doublets or neurons during the harmonized analysis above to represent the output of the first tier and input to the second tier. In this context, we trained another set of five neural network models (see Methods and Supplementary Table 6). A neural network model with one hidden layer (256 nodes) and an SGD optimizer showed the best performance (overall accuracy of 80%) and was selected for further work. Using the raw (normalized) data from each of the six original studies as an independent input to the two-tiered model, we found that it showed strong performance in identifying the neuronal sub-types in five of the six studies (Sathyamurthy, Hayashi, Haring, Rosenberg, and Zeisel) (Supplementary Fig. 11). The model may have shown poor performance for the sixth dataset (Baek) due to its very small size and minor contribution to the overall training data and to the neuron training data, as we observed a relationship between the fraction of correct predictions and the contribution to the training data in the context of all cell types (Supplementary Fig. 11). These results must be interpreted with caution because each of these datasets were included in the overall training data which could lead to artificially high performance.

As a final performance test of the two-tiered model, we applied it to spinal cord nuclei from a completely independent dataset that was not included in the integration or model training. As the model was trained on different data, overfitting is not a concern on this dataset, so these results are indicative of real-world performance on independent data. Nuclei were isolated from the lumbar spinal cords of four adult mice, sequenced using 10x Chromium, clustered using Seurat, and marker genes were identified for each cluster (Fig. 6d and Supplementary Fig. 13). 90% of nuclei (out of 28,584 total) were in clusters that could be assigned a cell-type label based on marker gene expression (known clusters). In cases for which labels could not be confidently assigned (10% of nuclei, unknown clusters), a placeholder name was given (Supplementary Fig. 13). We performed classification of all nuclei from the independent dataset that passed quality-control thresholds (Fig. 6c) in an analysis that took less than thirty minutes of computational time (~20 min for Tier 1 and less than one minute for Tier 2). We found that 90% of nuclei from known clusters were accurately classified by the two-tiered model (Fig. 6f known + accurate). We next considered how this model performed upon the classification of nuclei from the challenging unknown clusters that could not be identified based on marker genes. Surprisingly, we found that 28% of unknown nuclei could be identified with the two-tier classification model (Fig. 6f unknown + identified). Thus, the two-tiered model surpassed the ability of experienced users to identify spinal cord cell types.

Of note, several cell types were not expected to be present in the independent dataset, including Schwann cells, peripheral glia, and meninges 2 (based on the surgical dissection method used that did not include spinal roots or outer layers of meninges) and including PGC, Excitatory-7, and Excitatory-17 (based on the lumbar region and adult age that was used). As expected, these cell types were not predicted by the two-tiered model. There were also several cell types that were not classified as expected. In particular, several mid/ventral cell types were not detected in the independent dataset while two ventral clusters (Excitatory-31 and Inhibitory-27) were over-represented (Fig. 6 and Supplementary Fig. 13). This may reflect a training dataset that is not large enough to train a model that distinguishes closely related cell types, that small clusters are not modeled as well, and that some mid/ventral clusters are defined partly by early postnatal gene expression contained within the harmonized analysis but absent from the independent adult dataset.

These results establish a two-tiered model based on label transfer and a neural network as an effective approach for the computational classification of single-cell sequencing data, even in the context of the finely separated populations of spinal cord neurons. The neural network model was at least as accurate as other methods such as Seurat-based clustering and high-content in situ hybridization and was orders of magnitude faster. In addition, it can standardize spinal cord cell type classification so that a unified and harmonized set of cell types can be identified and studied consistently between datasets, biological conditions, and laboratories throughout the field.

### SeqSeek: a community resource for analyzing and classifying spinal cord cell types

Finally, we have developed an online resource for spinal cord single-cell data, SeqSeek (available at seqseek.ninds.nih.gov). This resource includes user-friendly tools to search gene expression across spinal cord cell types using single genes or gene lists (SeqSeek Genes), to compare gene expression between clusters or groups of clusters (SeqSeek Cells), and to access the SeqSeek algorithm for cell-type classification (SeqSeek Classify, also available on our Github repository https://github.com/ArielLevineLabNINDS).

## Discussion

For the field of spinal cord biology to build upon the incredible promise of single-cell technologies, it is critical to establish a standard set of cell types. Here, we leveraged and expanded upon the previously published single-cell sequencing studies of the postnatal mouse spinal cord to define 84 types of spinal cord cells. We present a harmonized atlas of these cell types; a validated combinatorial panel of markers to facilitate their study either in vivo, in tissue sections, and in vitro cell culture; putative embryonic lineages for each cell type; computational resources for classifying spinal cord cells based on transcriptomics; and a web-based resource, SeqSeek, to allow the community to interact easily with and explore single cell spinal cord data. This work establishes a common framework that will serve as a powerful resource for the field and facilitates the discovery of biological features of spinal cord cell types. As an example, we identified major differences between dorsal and ventral neuron types in their cluster relationships and in plasticity gene signatures, highlighting the primary role of spatial location in the organization of the mammalian spinal cord.

The first key consideration is whether the cell types of the atlas are correct biologically or whether they are confounded by technical issues contributed by the original studies or analysis choices that we made here. For example, it is possible that integrating these studies would obscure important biological differences between them or that merging early postnatal and adult datasets would blur proper cell type descriptions. In the absence of a commonly accepted standard set of spinal cord cell types, it is impossible to answer this question completely. However, several pieces of evidence support the accurate description of spinal cord cell types. First, highly reliable clusters were identified based on four independent integration methodologies – Seurat V3, Harmony, Conos, and LIGER – suggesting that these clusters represent the underlying biological reality of cell types. Second, these clusters correspond well with prior gene expression analysis of the postnatal spinal cord including many classic and well-established marker gene studies as well as three independent single nucleus sequencing datasets that were not included in the harmonized clustering: an independent dataset that we clustered separately and used to test the SeqSeek Classify algorithm, and two recent studies that used different analysis strategies but found similar markers to the harmonized set^8,9^. Third, and most importantly, this atlas does not rest only on select studies or on computational approaches that would be subject to the biases of particular tools and parameter choices. We performed high content in situ hybridization to test the validity of predicted expression profiles in the full transverse view of adult lumbar spinal cord tissue. In a few instances, this data differed from the harmonized sequencing data (for example in Excit-3 and Excit-4) which may reflect differences in developmental patterns. However, we validated the vast majority of predicted expression patterns from the harmonized atlas and the resulting data provided the most extensive characterization of cell types, their prevalence, and their spatial distribution in the postnatal spinal cord.

In addition to serving as a powerful reference resource, what new biological information can this study reveal? One of the most striking findings was the difference in cell type organization between the dorsal and mid/ventral regions of the spinal cord, both in cluster relationships and in general molecular trends. Dorsal clusters are distinct from each other with clearly separated individual cell types that can be grouped loosely into families. These cell types are located at greater distances from each other in principal component/UMAP space, have higher measures of robustness (such as co-clustering frequency and silhouette score), and can be reliably distinguished by machine learning algorithms or in tissue with combinatorial marker genes. In contrast, ventral clusters are much more similar to each other, with close or overlapping distributions in principal component space and overlapping gene expression patterns. Ventral neuron cell types may be organized at a second, nested level of spatial trends that overlay embryonic lineage-defined cell types: a Pou6f2-Esrrg trend along the dorsal-ventral axis and a Nfib-Zfhx3/4 and birthdate trends along the medial-lateral axis, consistent with a recent report^108^. It is not yet known what these differences between the dorsal and mid/ventral spinal cord may signify, but an exciting possibility is that discreet versus overlapping sets of cell types would give rise to different network computational properties^109^.

Related to these overall differences in cell type relationships, the dorsal and ventral regions of the spinal cord displayed broad molecular differences from each other that drove the primary bifurcation amongst spinal interneurons/projection neurons (non-motoneurons or CSF-cN neurons) in our cluster dendogram analysis, even before excitatory and inhibitory neuron types separated from each other. Differential gene expression and gene ontology analysis revealed that learning-related genes are enriched in the dorsal horn while structural stability-related genes are enriched in the ventral horn. Within the dorsal horn, this included both broadly expressed genes such as Camk2a (CAMK2α) as well as cell type-specific genes such as Prkcg (PKCγ)^31^. Within the ventral horn, this included components of perineuronal nets (which are thought to restrict plasticity and had been previously detected in the ventral spinal cord^110,111^) such as Tnr (Tenascin-R), as well as adhesion molecules such as Sdk1 and intracellular signaling components such as Ptpn5 (STEP) which de-phosphorylates CAMK2α, NMDA receptors, and ERK kinases^33^. This raises the intriguing possibility that meta-plasticity trends govern spinal cord circuits to facilitate learning in dorsal regions, where central sensitization^112^, wind-up^113^, and long-term potentiation and depression^114–116^ have been observed and may underly chronic pain states^117,118^. In contrast, the ventral horn may be stabilized to restrict certain plasticity mechanisms from altering core locomotor circuits. We had previously noted differences in the robustness of dorsal and ventral cell type clusters^1^ and a similar trend of overlapping ventral cell types was observed in the neonatal spinal cord^4^. Building on these preliminary findings, the scope of the harmonized analysis here afforded a much deeper characterization of cell type relationships and robustness, the validation of these molecular distinctions in tissue, and through machine learning. This work also led to the surprising discovery of gene expression signatures for plasticity in the dorsal horn and for structural stability in the ventral horn.

This work also provides a broad view of the relationships between embryonic lineage domains and their mature neuronal progeny. For the past thirty years, the cardinal classes of spinal cord progenitors have been used as a framework to classify spinal cord cell types, particularly within the ventral horn of the spinal cord^13,15,16,119,120^. However, it has been challenging to relate these domains to cell types defined in the adult by function, connectivity, or electrophysiology and it has therefore been unclear how these perspectives on cell type intersect and which perspective is the most useful for linking spinal cord neurons to behavior. Here, we co-integrated an embryonic (e9.5–e13.5) spinal cord sequencing dataset^7^ with our harmonized analysis and identified putative lineages of many postnatal cell types. These relationships must be tested experimentally in future studies but, if true, they reveal two intriguing trends in spinal cord cell type organization. The first trend is that cell types within the family structure that we described generally shared common embryonic lineages, suggesting a developmental basis for the group resemblances. For example, the deep dorsal horn excitatory neurons can be divided into two families, with Excit-21 through Excit-25 in one family that is likely derived from dI5 precursors and Excit-26 through Excit-29 in a looser family that includes dI1/2 and V0 precursors. The second trend is that many cell types were derived from multiple lineage domains. For example, dI3.3 and dI3.4 were found together with dI5.3 and dI5.4 in a long trajectory toward the majority of dorsal excitatory neurons. It is already known that the adult cell types of Ia inhibitory neurons and CSF-contacting neurons each have dual origins^43,45,121^, suggesting that this could be a common occurrence. Perhaps there isn’t a simple but rigid logic by which cardinal classes mature and differentiate into distinct and refined populations. Rather, a complex process may operate in which there is both divergence and convergence in the relationships of developmental and mature cell types, with influences such as birthdate, cell body location, connectivity, or activity-dependent maturation playing important modulatory roles. By providing a broad perspective on developmental and mature cell type similarities, this work suggests that there are multiple schemes that guide the differentiation of spinal cord neural precursors into the highly refined and diverse array of neurons that mediate adult behavior.

On the analytical side, this work is among the first practical applications of automated classification for large and complex single-cell datasets from neural tissue. A wide range of cell annotation approaches have been described recently but it is not yet clear which methods will work best for each type of data^23,122–125^. A comparative analysis of automated classification approaches across diverse datasets found that SVM and neural network models showed the best performance on the Allen Brain Atlas dataset of 92 neuronal cell types–a dataset similar in scale and complexity to the harmonized analysis here^125^. This analysis also found that performance depends partly on the number of cell types and the complexity (the relatedness between clusters) of a dataset, similar to what we observed. Here, we found that a two-tiered model that incorporates label transfer and a neural network displayed excellent performance in the computationally challenging task of classifying cells and nuclei into the 69 fine resolution neuronal cell types of the spinal cord. In the future, larger spinal cord single-cell datasets will be available and the neural network model that we presented here can be refined and improved. Specifically, larger training datasets may facilitate classification of closely related mid/ventral neuronal populations; region or sample age-specific training datasets may reduce the number of cell types that cannot be detected; and generative models may be used to enhance training on rare cell populations. As this work proceeds, we expect that increasingly powerful neural network models will be developed that allow rapid, accurate, and standardized classification of all spinal cord cell types directly from raw sequencing data. This could be done by individual users with downloadable models or through the development of a spinal cord single-cell data commons that could continuously refine the models and provide classification analysis through a cloud-based platform, similar to what has been proposed for the Human Cell Atlas^126^. A forthcoming study aims to partially address these challenges. Theis and colleagues propose a method called *single-cell architectural surgery* that uses transfer learning to map query datasets onto a reference, simultaneously contextualizing the query while updating the reference. This allows for decentralized reference building without the sharing of raw data, which could further increase the effectiveness of neural network-based classifiers^127^.

There are several notable limitations to this study. Most specifically, this analysis is limited in scope to RNA expression in the postnatal mouse spinal cord and reflects a merged study of multiple time points. As more data become available from studies that include more specific regions of the spinal cord, more biological conditions, more developmental stages, more species, more specific cellular features, and more -omics modalities, we anticipate that this work will reveal exciting insights from single-cell data. Future work could incorporate genetic lineage tracing to test developmental origins for postnatal cell types^2^, could track cell-type-specific changes in different biological conditions^8,9,128^, or could focus deeply on specific spinal cord regions and cell types^10,11,39,40,77,129^. Relatedly, the in situ hybridization experiments here are also limited in scope, being specific to the adult lumbar spinal cord. The failure to detect several genes from the harmonized analysis could reflect that these genes are no longer expressed at the adult stage or lumbar region that we analyzed, that the cell types themselves are not present (being transiently found in early postnatal stages or only in other spinal cord regions), or technical issues. We caution users of the SeqSeek resource to keep this in mind when examining individual data points.

A second notable caveat that is common to most single-cell sequencing experiments is that this analysis is population-based. Data is captured from thousands of individual cells, but the rate of false-negative data in each cell and the requirement for statistical power necessitates analyzing many cells of each type and considering population-level shared patterns. It is likely that by emphasizing common patterns, this analysis underrepresents true biological variability, including noisy gene expression and continua of cell types. For example, three very different methods – single-cell data clustering, multiplexed in situ hybridization, and an artificial intelligence neural network – all showed a relatively weak ability to classify ventral cell types into discrete types and a relatively strong but still imperfect ability to classify dorsal cell types. We propose that this reflects some technical limitations but also a fundamental complexity and diversity in how gene expression is controlled within individual cells and in cell-type populations.

Third, as future datasets and technologies become available, we anticipate an explosion of single-cell data and the opportunity to periodically supplement, revise, and refine the work presented here. In this context, the harmonized atlas is both a work in progress that will continue to evolve over time and the gold standard that we have now as the most comprehensive and validated resource available for the mammalian spinal cord.

Finally, it is crucial to note that single-cell/nucleus profiling, particularly single-cell/nucleus RNA sequencing, produces one perspective on cell types and it is not yet clear how this will relate to other core cellular features such as circuit connectivity, electrophysiology, and behavioral function. Re-considering the very definition of a cell type and identifying the most useful system for classifying cells is now a fundamental task in understanding nervous system function. We expect that in each tissue, indeed in each region of each tissue, there may be different organizing principles of cell types. In that context, the work here provides a comprehensive atlas of spinal cord transcriptomic cell types that can be used as a framework to compare with other cellular features.

This work brings together the first single-cell studies of the post-natal mouse spinal cord to create a standard reference set of spinal cord cell types. It will (1) serve as a unifying resource and nomenclature for the field, (2) provide a validated and combinatorial set of markers that can be used to translate this rich sequencing data back into tissue-based studies, (3) be a template for the computational analysis of single-cell data from complex neural tissue, and (4) facilitate the community-wide use of single-cell data through a web-based resource. We hope that this work will facilitate the design and interpretation of cell-based studies of behavior and will open up opportunities for many discoveries.

## Methods

### Mice

Animal experiments were performed in accordance with institutional guidelines and approved (protocol #1384) by the National Institute of Neurological Disorder and Stroke’s Institutional Animal Care and Use Committee. An even balance of male and female mice that were 9 weeks old and of mixed C57BL/6 J and BALB/cJ background were used for single nucleus sequencing (four mice) and validation studies (two groups of three mice).

### Published data acquisition

Published data were downloaded from the NCBI Sequence Read Archive (SRA). Raw datasets were used instead of investigator-provided count matrices so that we could align all sequences to the same genome and apply uniform data filtering. All raw datasets were pre-processed using technique-specific pipelines. For data from Sathyamurthy et al. (DropSeq, GEO: GSE103892, SRA: SRP117727), data were downloaded in fastq format from SRA. A count matrix was created following the steps in the McCarroll lab DropSeq cookbook^130^. For data from Hayashi et al. (GEO: 108788, SRA: SRP128071), Zeisel et al. (SRA: SRP135960), and Baek et al. (GEO: GSE130312), 10X sequence data were download from SRA in BAM format then converted to cell ranger-compatible fastq files using the 10X-provided bamtofastq tool. Count matrices were created using the 10X cell ranger count tool. Data from Haring et al. (C1 Fluidigm, GEO: GSE103840, SRA: SRP117627) were downloaded from SRA. Each cell had its own fastq file for a total of 1545 files. We followed the UMI tools single-cell tutorial to remove the UMI and process the sequences (https://github.com/CGATOxford/UMI-tools/blob/master/doc/Single_cell_tutorial.md). For the Rosenberg et al. data (SplitSeq, GEO: GSE110823, SRA: SRP133097), data were downloaded in fastq format. Count matrices were made using the split-seq-pipeline tool developed by the Seelig Lab (https://github.com/yjzhang/split-seq-pipeline). The STAR alignment tool within cell range (v020201) was used to align the sequences from each dataset to a reference genome that was custom built to include all introns and exons, based on mm10, GRCm38 updated on 2016-01 (NCBI: GCA_000001635.6). Reference: https://support.10xgenomics.com/single-cell-gene-expression/software/downloads/2.0/. Genome: https://cf.10xgenomics.com/supp/cell-exp/refdata-cellranger-mm10-1.2.0.tar.gz.

### Merged analysis and integration

Count matrices for each dataset were merged to obtain the full data file and we then applied uniform data filtering across the merged file. We analyzed all cells and nuclei with at least 200 detected genes (to exclude low quality or empty barcodes) and with less than 5% of transcripts being mitochondrial (to exclude lysing cells or mitochondria-nuclei doublets). This yielded over one hundred thousand total cells/nuclei. Of note, by starting with the raw data and setting relatively relaxed thresholds for data inclusion, we analyzed more cells/nuclei from several of the original studies than were analyzed in the corresponding published datasets.

The merged data were analyzed using Seurat v3^23,131^. The main integration was performed using Seurat version 3.0 Standard Workflow (CCA) Integration^131^ such that data were LogNormalized and scaled to 10,000 counts. Highly variable genes were found using the default var.mean.plot method, a mean cutoff at 0.0125 and 3 and a dispersion cutoff at 0.5. The data were then scaled with a linear model and while regressing out the number of counts and the percent mitochondria. The top 100 PCs were calculated. Integration anchors were calculated using 20 PCs and used to integrate the data. This integration was compared to three independent methods (Harmony^25^, Conos^26^, and LIGER^27^). In the case of Harmony integration, SCTransform normalization was used prior to performing the integration. Annotations from Seurat integration were then overlapped on the integrated UMAP projections obtained from other integration methods and the cell type clusters were compared for reproducibility. The neurons specific population of cells was pulled out from Seurat integration results and integrated using Harmony integration, in order to compare neuronal subclasses from two integration protocols.

### Clustering

Clustering was performed in three phases on (1) all cell types, (2) all neurons, (3a) presumptive ventral neurons, and (3b) motor neurons. For phase 1, data integration was performed by study, 2,000 highly variable genes were detected, and the most significant principal components were identified by elbow plot and manual inspection of the contributing gene lists and 28 PCs were used for clustering. To select cluster resolution, a range of values were tested from 0.2–8 and cluster evolution or clustree plots were used to determine when cluster splitting stabilized, and resolution 1.2 was selected. For phase 2, raw data from all cells in neuronal clusters was used, re-scaled, re-normalized, and re-integrated, the top 4000 highly variable genes were detected and the top 40 PCs were selected (using the approach described above as well as statistical jackstraw and elbow plot analysis, see Supplementary Fig. 4). In analyzing neuronal diversity, we favor an approach of using a higher resolution, as long as clusters are still robust, and then examining whether pairs of clusters should be merged, as we described in a recent paper^132^. Here, the range of resolutions were examined by (1) the range of average silhouette scores for the clusters, (2) visual inspection of UMAP cluster distribution, and, most importantly (3) comparison of cluster markers with known markers and with known co-expression patterns in the literature. (Supplementary Fig. 4). The third phase of targeted sub-clustering was done because mid/ventral and motoneuron sub-types did not separate well in preliminary neuron analysis. Indeed, the robustness scores for mid/ventral cell types were very low until they are analyzed in a focused principal component space (Supplementary Fig. 2). For phase 3a, presumptive ventral neurons were identified by markers and by coalescence on UMAP into a central blob and for phase 3b, motorneurons were identified by expression of classic markers (Chat, Isl1, Prph). In each case, the procedures described above were used to sub-divide these cell types and the following parameters were used: 3a: 40 PCs, resolution 4; 3b 7 PCs, resolution 0.6.

For all three phases, each cluster was analyzed for candidate marker genes and excluded if the cluster met either of the following criteria. Clusters were considered low-quality if they had fewer than three significant markers relevant to cell type, particularly if they showed very low nGene. Clusters were considered doublets if they had significant markers for multiple unrelated cell types and a barnyard plot of the top ten markers of each cell type showed that individual cells in the cluster displayed both sets of markers. For all three phases, we used the following method to determine whether candidate pairs of clusters should be merged: a dendrogram based on mean gene expression and UMAP location were used to systemically identify closely related clusters and we then probed for differential gene expression (for example, see Supplementary Fig. 4). Pairs with fewer than three genes enriched in each cluster (six total) were merged unless a classic marker gene from the literature was one of five differentially expressed genes. Cell type annotations for the non-neuronal cell types were based on the presence of well-established marker genes (Supplementary Table 1) and on the gene expression patterns in the Allen in situ hybridization database (for meningeal, ependymal, Schwann cell, and peripheral glia clusters).

The meta-data (and associated final cell labels) are available in Supplementary Table 5.

### Cell type relationships, comparison with prior studies, and differential gene + GO analysis

To examine the relationship between the 69 neuronal clusters in the harmonized analysis, the centroid of each cluster was calculated by grouping the cells by their labels and determining the mean of each PC. Then, the pairwise Euclidean distance between each cluster was calculated using 50 PCs. This was passed to the stats::hclust function using method = “complete”. The final dendrogram was plotted using the graphics::plot function.

To examine the distribution of the original Haring and Sathyamurthy clusters amongst the harmonized clusters, the frequency of each pair-wise combination of original and harmonized clusters was counted. These data were then pivoted to wide form to produce the matrix with harmonized clusters along the x-axis and original clusters along the y-axis. Finally, the data was row-normalized, so that the color represents the fraction of the original label occurring in each harmonized cluster.

To examine the distance between the original Haring and Sathyamurthy clusters in harmonized PC space, the pairwise distance between the centroids of the original clusters was calculated as above. Small distances, representing close clusters, are displayed with hot colors, while large distances, representing far apart clusters, are displayed with cold colors.

To examine the correlation between PC distance and the expression of the 500 most highly variable genes in the harmonized data, the average expression of these genes was calculated for each original cluster, which yielded two matrices: one a gene by cluster matrix of the Haring data, and the other a gene by cluster matrix of the Sathyamurthy data. The correlation of gene expression in each cluster between these matrices was calculated using the lineup::corbetw2mat function (CRAN version 0.37.11). These correlation scores were then plotted against the PC distances calculated above. Linear regression with 95% confidence intervals is shown.

Differential gene expression for the dorsal/ventral and excitatory/inhibitory analysis was performed using the ROC test in Seurat, with genes in >30% of each class and with a log FC > 0.25. Genes with a ROC > 6 were compiled into lists and analyzed using default parameters in GO DAVID, with molecular function and biological process GO terms selected, as well as KEGG pathway terms.

### RNA In situ hybridization, Immunofluoresence, and WFA staining

For high content RNA in situ hybridization, 14 µm fresh frozen spinal cord sections from segment L4 were placed on Leica Apex slides and sets of 97 RNAScope HiPlex probes were used (Supplementary Table 3) from ACDBio, according to the manufacturer’s instructions. Images for each set were registered using RNAscope HiPlex Image Registration Software and brightness/contrast were adjusted using Adobe Photoshop. Counting of cells was done by first using a general class marker in each panel of probes (such as Slc17a6 or Slc6a5) to focus counting on neurons of a particular neurotransmitter status and by considering one region at a time (dorsal, mid, or ventral). In addition, the following guides were used. Set 1: All Chat+ cells in any laminae. Set 2: Any dorsal cell that expressed any of Cpne4, Maf, or Prkcg. Set 3: Any cell in the dorsal horn with any of Slc17a6, Rreb1, Reln, or Car12. In addition, Gbx2 cells were counted separately amongst any cell in the deep dorsal horn with Slc17a6. Set 4: Any cell in the dorsal horn with any of Col5a2, Enpp1, Sox5, Tac1, Tac2, Nmu, Megf11, Mdga1, Pmfbp1, or Onecut2. Set 5: Any cell in laminae 1-4 with any of Slc6a1, Gad2, or Kcnip2. Set 6: Any cell in the dorsal horn with any of Mlxipl, Pdyn, Gal, Npy, Qrfpr, Sstr2, or Rspo3. Set 7: Any cell in laminae 4-6 with any of Slc17a6, Adamts2, Lmx1b. Set 8: Any cell in laminae 4-6 with either Slc6a5 or Gad2. Set 9: Any cell in laminae 6-8 with Slc17a6. Set 10: Any cell in laminae 6-8 with any of Pax2, Slc6a5, or Gad2. The number of cells counted in each set are listed in Supplementary Table 3 and were from one section per animal, though multiple sections per animal were inspected for expression pattern consistency. Sections from three animals (2 male and 1 female or 2 female and 1 male) were counted for each set. For Fig. 3c, e, V2 RNAScope probes were used (also from ACDBio, according to the manufacturer’s instructions). For immunofluorescence and lectin staining, animals were perfused, and 50 µm sections of the frozen section were cut and stained. To detect CAMK2α, Millipore 905-532 was used (1:500). For perineuronal net WFA-lectin staining, fluorescent lectin (Vector Laboratories, FL-1351-2) was used according to the manufacturer’s instructions.

### Single nucleus sequencing

Nuclei were obtained as previously described^133^ and were processed for single-cell sequencing using the 10X Genomics Chromium Single Cell 3′ Kit (v3 chemistry) and sequenced at a depth of approximately 50,000 reads per nucleus. Clustering was performed as described above and cluster identities were determined using the combinatorial marker code in Table 1 where possible (known clusters). Clusters that could not be identified in this manner were analyzed for neurotransmitter status and given a placeholder identification (unknown clusters).

### Computational classification

#### Label transfer

Label transfer analysis was performed using Seurat v3(.1.5). For both coarse cell types and clean neurons, 10% of cells were withheld as the query dataset, whilst the remaining were used as the reference dataset. Broadly, label transfer consists of two steps. First, the transfer anchors are identified using the FindTransferAnchors function. Second, these anchors are then used to transfer cluster labels to the query dataset with the TransferData function.

For label transfer of coarse cell types, FindTransferAnchors was called with reduction = “pcaproject”, dims = 1:28, and npcs = NULL to project the previously calculated PCA onto the query data using the same dimensions as were used in clustering the reference data. Transfer data was also called with dims = 1:28 for the same reason.

Label transfer of clean neurons was performed in a two-step process. First, all cells in mid- or ventral-clusters were grouped as one cluster. Then, the dorsal-clusters were transferred along with one mid/ventral cluster. Second, those cells classified as mid/ventral were labelled using only neurons from mid- or ventral-neuron clusters. In each case, a new reference object was created from the appropriate cells – all neurons for step 1 and mid-/ventral-neurons only for step 2–via integration, as previously discussed in the Merged Analysis and Clustering section. Label transfer was run as described for coarse cell types, with the exception that dims = 1:100 was set for all neurons, and dims = 1:30 was set for mid-/ventral-neurons.

In the final two-tier analysis, label transfer was performed as discussed for coarse cell types. Any cells labelled neuron, motor neuron, or doublets were passed to the neural network for further classification. The decision to include doublets for further classification was founded on the observation that a non-trivial number of neurons were misclassified as doublets at the coarse cell-type level.

#### Support vectror machine

Support vector machine analysis was performed using scikit-learn version 0.22.2.post1. Count matrices were taken from the default Seurat RNA assay count slot as sparse matrices. Cluster labels were numerically encoded with LabelEncoder(). To preserve sparsity for reduced training time, these counts were scaled with MaxAbsScaler(copy = False). As LinearSVC() is known to be faster and more scalable than SVM(kernel = “linear”), it was selected for use (https://scikit-learn.org/0.22/modules/svm.html#svm-classification and https://scikit-learn.org/0.22/modules/generated/sklearn.svm.LinearSVC.html#sklearn.svm.LinearSVC). As the number of samples was significantly greater than the number of features, the dual parameter was set to “False”. Finally, to help ensure convergence, the max_iter parameter was increased from the default of 1000 to 10000. This pipeline achieved an overall accuracy of 80% on the validation data. Though this performance could likely be improved by hyperparameter tuning, given the performance of alternative models, the support vector machine was not selected for further use.

#### Neural networks

Count Matrices were taken out of the default Seurat RNA assay count slot as sparse matrices. Genes with no counts were dropped. The counts were log x+1 transformed then scaled by the maximum number of counts for any gene in a cell. The data were converted into TensorFlow sparse tensors for input into neural networks define via the Keras interface to TensorFlow. Hyperparameters were initially set to default values, with a network structure consisting of direct connections between the input and output nodes. This simple linear model was the baseline. We added additional layers from 1 to 4 hidden layers, at various widths from 16 nodes to 512 nodes in a layer. The optimizer we switch from the default “Adam” optimizer to singular gradient descent (sgd). L1, L2, and dropout regularization were attempted. Additionally, various batch sizes were tested. Initially, networks trained for coarse analysis used a batch size of 128 to speed training. Whereas the training was faster, validation accuracy improved by around 5% when we lowered the batch size to 32. No additional improvement was seen at a batch size of 16, so the batch size was set to 32 for the rest of the study. In general, we used the learning curves to guide the changing of hyperparameters^134^.

For the analysis of coarse cell types (Fig. 5a), a model with two hidden layers of 512 nodes each and L2 regularization was used. For the analysis of the neuronal subtypes (Fig. 5b), seven models were tested: (1.1) a linear model with no regularization (1.2) a linear model with L2 regularization (learning rate 0.001) (1.3) a neural network with two hidden layers of 512 nodes each (1.4) an ensemble-like neural network with one hidden layer (128 nodes and L2 regularization) and two hidden layers that were concatenated, (1.5) a neural network model with three hidden layers (512, 256, 128 and L2 regularization on the 512 nodes hidden layer (1.6) a neural network model with 3 layers (128, 128, 128 and L2 regularization on the first hidden layer) and (1.7) a linear model with no regularization with an SGD optimizer. Interestingly, the baseline model had the largest validation accuracy. Since the training accuracy is 100% as compared to 85% in the validation set, the model is clearly overfitting the training data. Adding regularization helped to lower the gap between the training and validation accuracy, but the overall validation and test accuracies are still lower suggesting that the overtrained model will perform better on unseen data. Additional work to improve this model is needed and adding more data from further experimental studies in the future will help improve the validation accuracy. For the analysis and training of neurons and doublets together (Tier 2), five models were tested: (2.1) a linear model with no regularization (2.2) a linear model with L2 regularization (2.3) a neural network model with one hidden layer of 128 nodes (2.4) a neural network model with one hidden layer of 128 nodes and SGD optimizer, and (2.5) a neural network model with one hidden layer of 256 nodes and SGD optimizer. The final model (2.5) was selected for Tier 2.

In the analysis of unknown clusters (Fig. 5f), individual nuclei were identified if (1) they were from an unknown cluster and were classified into a harmonized true cell type (not junk or doublets) and (2) at least 80% of the total nuclei from their cluster of origin were classified into the same single harmonized cell type.

For the two-tier classification method, Tier 1 was run on an Apple MacBook Pro Core i9 2.3 GHz, 32 GB, 1TB Radeon Pro, and took approximately 20 min. Tier 2 was run on Google Colab (the CPU was not guaranteed but was Intel® Xeon® CPU ^@^2.20 GHz 2 CPU, 13 GB RAM, 107 GB disk. The runtime was 16 s to read in 14 MB of test data and 1 s to run the neural network.

Supplementary Analysis Notes

1. Consideration of the decision to include formalin and rotarod experimental samples as part of the Sathyamurthy et al. dataset: To ensure that the inclusion of these samples would not bias the clustering or the gene expression patterns, we performed the following analysis. First, we analyzed the neuronal cluster distribution of each experimental condition. Overall, nuclei from the formalin and rotarod conditions accounted for 11.0% of the total neuronal cells/nuclei and also represented 11.1% (±0.7 standard error) of each cluster. The only clusters that had >16% contribution from an experimental condition were in the mid/ventral regions of the spinal cord (in which the Sathyamurthy dataset is somewhat overrepresented in general). Therefore, we concluded that the cluster contribution is not biased by experimental conditions. Second, we analyzed whether the gene expression of the clusters could be biased by the inclusion of the experimental conditions in two ways. We used the original set of nuclei from the Sathyamurthy dataset and tested whether any genes were differentially expressed between the experimental conditions. The only gene that was significantly different (ANOVA, corrected *p*-value < 0.05) was the immediate early gene *Fos*. This is not unexpected because the formalin and rotarod samples in the Sathyamurthy dataset were collected five minutes after the intervention, a very short time window that makes major changes in gene expression or cell composition exceedingly unlikely. Next, we performed a more refined analysis and compared genes that were differentially expressed (Wilcox test) *within* each cluster, between nuclei from an experimental condition and all naïve cells/nuclei from any dataset. Although there were genes that were different in many of the clusters, this likely reflects the general differences in sample age and technique between the studies. Only one differentially expressed gene was an immediate early gene (*Homer1a* was found in a higher fraction of cells/nuclei from the experimental condition in cluster Inhib-8 but also had a lower expression level per cell/nucleus). Therefore, we concluded that the gene expression profiles are not biased by the inclusion of these nuclei. In fact, the broadly elevated levels of immediate early genes in the Haring and Zeisel datasets (Supplementary Figs. 1 and 3) likely washes out any small effect of the behavioral conditions from the Sathyamurthy dataset.
2. Examination of cell vs. nuclei gene expression differences: To explore systematic differences in the gene expression profiles between studies that used cells and those that used nuclei, we performed the following analysis. First, we note that overall, the number of genes per cell/nucleus, the expression of immediate early genes, and the expression of stress-related genes were all different based on technique (Supplementary Fig. 3). In examining cluster composition, we found that only studies that used nuclei contributed to MN-alpha, MN-gamma, or PGC cells as well as Excit-7 and most ventral neuronal clusters (Supplementary Table 6). We next compared differential gene expression *within* each neuronal cluster for studies that used cells or nuclei (Wilcox, minimum FC = 0.25, minimum percent of cells expression = 10%), selected the top 30 genes ranked by adjusted *p*-value, and removed duplicates. We found that 484 unique genes were differentially expressed and of these, 482 (99.6%) were enriched in cells compared to nuclei. GO analysis revealed that all of the enriched gene annotation clusters were associated with basic cell metabolism terms such as the ribosome, metabolic pathways, and proton transport (Supplementary Table 6). To further probe genes that were enriched in nuclei compared to cells, we sorted all significant genes in each cluster by the average log fold change, selected genes with a value > 2 (nuclei > cells), and identified a list of 10 protein-coding genes and 3 lncRNAs (Supplementary Table 6). In summary, the major differences that we observed were that ventral cell types were mainly detected in studies that used nuclei compared with cells, (which may reflect a differential vulnerability of ventral cells to stressful cell dissociation methods), and that general metabolism genes were enriched in studies that used cells (which may reflect increased detection of genes that have low levels of expression).

### Reporting summary

Further information on research design is available in the Nature Research Reporting Summary linked to this article.

## Supplementary information

Supplementary Information

Description of Additional Supplementary Files

Supplementary Movie 1

Reporting Summary

Supplementary Table 1

Supplementary Table 2

Supplementary Table 3

Supplementary Table 4

Supplementary Table 5

Supplementary Table 6

Supplementary Table 7

Supplementary Table 8

## Data Availability

The raw sequencing data generated in this study have been deposited in the NCBI database under accession code GSE158380 (https://www.ncbi.nlm.nih.gov/geo/query/acc.cgi?acc=GSE158380). In addition, the publicly available data utilized in this study are available at: Sathyamurthy: https://www.ncbi.nlm.nih.gov/geo/query/acc.cgi?acc=GSE103892 Hayashi: https://www.ncbi.nlm.nih.gov/geo/query/acc.cgi?acc=GSE108788 Zeisel: https://www.ncbi.nlm.nih.gov/sra/SRP135960 Haring: https://www.ncbi.nlm.nih.gov/geo/query/acc.cgi?acc=GSE103840 Rosenberg: https://www.ncbi.nlm.nih.gov/geo/query/acc.cgi?acc=GSE110823 Baek: https://www.ncbi.nlm.nih.gov/geo/query/acc.cgi?acc=GSE130312 Blum: https://www.ncbi.nlm.nih.gov/geo/query/acc.cgi?acc=GSE161621 Alkaslasi: https://www.ncbi.nlm.nih.gov/geo/query/acc.cgi?acc=GSE167597 Delile: https://www.ebi.ac.uk/arrayexpress/experiments/E-MTAB-7320/files) A searchable version of all processed data from the harmonized analysis is available www.seqseek.ninds.nih.gov.

## Code availability

The code associated with this study is available at https://github.com/ArielLevineLabNINDS. Specific code for the full SeqSeek classifier is available at DOI: 10.5281/zenodo.5081263. Code for the neuron neural network only is available at DOI: 10.5281/zenodo.5081268. Sample data for running the classifier is available at DOI: 10.5281/zenodo.5081266.
