# Author Correction: A harmonized atlas of mouse spinal cord cell types and their spatial organization

**DOI:** 10.1038/s41467-022-28698-7

**Published:** 2022-02-18

**Authors:** Daniel E. Russ, Ryan B. Patterson Cross, Li Li, Stephanie C. Koch, Kaya J. E. Matson, Archana Yadav, Mor R. Alkaslasi, Dylan I. Lee, Claire E. Le Pichon, Vilas Menon, Ariel J. Levine

**Affiliations:** 1 Division of Cancer Epidemiology and Genetics, Data Science Research Group, National Cancer Institute, NIH, Rockville, MD USA; 2 Spinal Circuits and Plasticity Unit, National Institute of Neurological Disorders and Stroke, NIH, Bethesda, MD USA; 3 Department of Neuroscience, Physiology and Pharmacology, Division of Biosciences, University College London, London, UK; 4 Department of Neurology, Center for Translational and Computational Neuroimmunology, Columbia University, New York, NY USA; 5 Eunice Kennedy Shriver National Institute of Child Health and Human Development, NIH, Bethesda, MD USA; 6 Department of Neuroscience, Brown University, Providence, RI USA

**Keywords:** Transcriptomics, Cellular neuroscience, Molecular neuroscience, Spinal cord

Correction to: *Nature Communications* 10.1038/s41467-021-25125-1, published online 29 September 2021.

The original HTML version of this Article did not link to Supplementary Tables 1 to 8. This has been corrected in the HTML version of the article.

The original version of this Article contained an error in the Results subsection entitled ‘Developmental lineages of postnatal spinal neuron populations’, in which a sentence incorrectly referred to Supplementary Table 6 instead of Supplementary Table 5. The incorrect sentence reads ‘In addition, the identity of the individual embryonic cells that were closest in PC-space to each cluster were also determined (Supplementary Table 6)’. The correct version states ‘(Supplementary Table 5)’ in place of ‘(Supplementary Table 6)’.

The original version of this Article contained an error in Methods subsection entitled ‘Published data acquisition’, in which one sentence omitted to cite Supplementary Table 7. The incorrect sentence read ‘Published data were downloaded from the NCBI Sequence Read Archive (SRA)’. The correct version adds ‘and are available in Supplementary Table 7’ after ‘(SRA)’.

The original version of this Article contained an error in the Methods subsection entitled ‘Clustering’, in which a sentence incorrectly referred to Supplementary Table 5 instead of Supplementary Table 7. The incorrect sentence read ‘The meta-data (and associated final cell labels) are available in Supplementary Table 5’. The correct version states ‘Supplementary Table 7’ in place of ‘Supplementary Table 5’.

The original version of this Article contained errors in the Methods subsection entitled ‘Supplementary Analysis Notes’. A sentence incorrectly referred to Supplementary Table 6 instead of Supplementary Table 2. The incorrect sentence read ‘In examining cluster composition, we found that only studies that used nuclei contributed to MN-alpha, MN-gamma, or PGC cells as well as Excit-7 and most ventral neuronal clusters (Supplementary Table 6).’ The correct version states ‘(Supplementary Table 2)’ in place of ‘(Supplementary Table 6)’. In addition, two sentences incorrectly referred to Supplementary Table 6 instead of Supplementary Table 8. The incorrect sentences read ‘GO analysis revealed that all of the enriched gene annotation clusters were associated with basic cell metabolism terms such as the ribosome, metabolic pathways, and proton transport (Supplementary Table 6).’ and ‘To further probe genes that were enriched in nuclei compared to cells, we sorted all significant genes in each cluster by the average log fold change, selected genes with a value > 2 (nuclei > cells), and identified a list of 10 protein-coding genes and 3 lncRNAs (Supplementary Table 6)’. The correct versions state ‘(Supplementary Table 8)’ in place of ‘(Supplementary Table 6)’.

These errors have been corrected in the PDF and HTML versions of the Article.

## Supplementary information

Supplementary Table 1

Supplementary Table 2

Supplementary Table 3

Supplementary Table 4

Supplementary Table 5

Supplementary Table 6

Supplementary Table 7

Supplementary Table 8

