# Author Correction: A harmonized atlas of mouse spinal cord cell types and their spatial organization

**DOI:** 10.1038/s41467-022-33998-z

**Published:** 2022-10-19

**Authors:** Daniel E. Russ, Ryan B. Patterson Cross, Li Li, Stephanie C. Koch, Kaya J. E. Matson, Archana Yadav, Mor R. Alkaslasi, Dylan I. Lee, Claire E. Le Pichon, Vilas Menon, Ariel J. Levine

**Affiliations:** 1 Division of Cancer Epidemiology and Genetics, Data Science Research Group, National Cancer Institute, NIH, Rockville, MD USA; 2 Spinal Circuits and Plasticity Unit, National Institute of Neurological Disorders and Stroke, NIH, Bethesda, MD USA; 3 Department of Neuroscience, Physiology and Pharmacology, Division of Biosciences, University College London, London, UK; 4 Department of Neurology, Center for Translational and Computational Neuroimmunology, Columbia University, New York, NY USA; 5 Eunice Kennedy Shriver National Institute of Child Health and Human Development, NIH, Bethesda, MD USA; 6 Department of Neuroscience, Brown University, Providence, RI USA

**Keywords:** Transcriptomics, Cellular neuroscience, Molecular neuroscience, Spinal cord

**Correction to**: *Nature Communications* 10.1038/s41467-021-25125-1, published online 29 September 2021

The original version of this Article contained an error in Fig. 3A, in which one label ‘Excit-21’ was inadvertently duplicated instead of the correct label ‘Excit-31’, and another label ‘Excit-32’ was inadvertently omitted, from the right hand side of the panel. This has been corrected in both the PDF and HTML versions of the Article.

