# Supplementary material for: A harmonized atlas of mouse spinal cord cell types and their spatial organization
Source: Nat Commun. 2021 Sep 29;12:5722. doi: 10.1038/s41467-021-25125-1 (PMC8481483; doi:10.1038/s41467-021-25125-1)
Supplement: Supplementary file 1 — Supplementary Information [file 41467_2021_25125_MOESM1_ESM.pdf]

Supplemental Figure 1

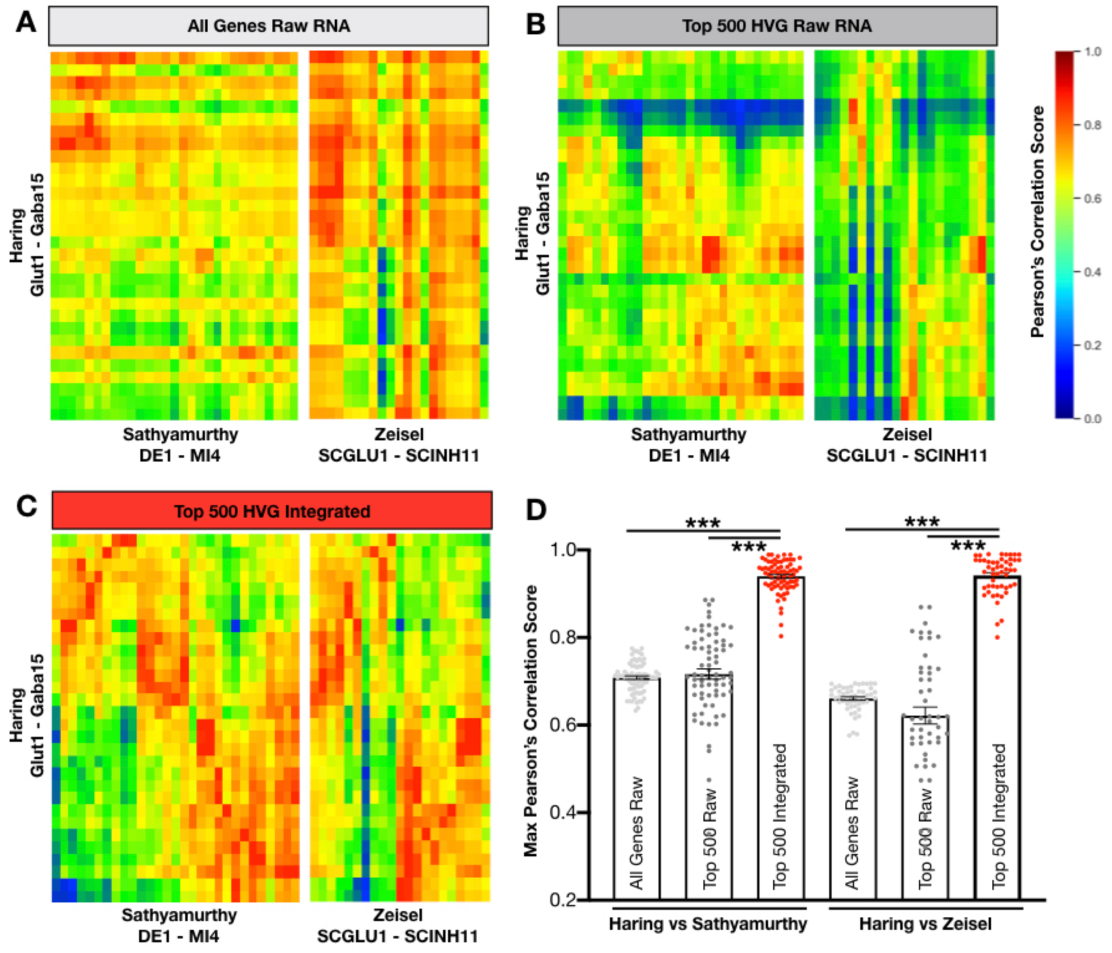

Supplemental Figure 1 (supplement for Main Figure 1). The correlation in gene expression between the original dorsal neuronal clusters in the Sathyamurthy et al., Haring et al., and Zeisel et al. studies. (A) Analysis was performed using the raw values for all genes, (B) the raw values for only the most highly variable genes (amongst neurons), (C) or the integrated values for only the most highly variable genes (amongst neurons). The original clusters from Haring et al. are shown in rows, proceeding from Glut1 – Gaba15. The original dorsal/mid clusters from Sathyamurthy et al. are shown in columns on the left, proceeding from DE-1 through DI-9, then ME-1, MI1-4. The original clusters from Zeisel et al. are shown in columns on the right, proceeding from SCGGLU1 – SCIN11. The Pearson's correlation score for each pair of clusters is shown, colored from blue = 0 through red = 1. (D) The maximum Pearson's correlation score obtained for each cluster, shown as dots for individual values (not replicates), and the mean +/- s.e.m. (bar and error bars) when the analysis for A, B, and C were performed. Comparison of the raw gene expression values between studies failed to identify clear relationships in clusters between datasets, while using the integrated values improved this significantly (*** is p<1E-15, unpaired t-test with Welch's correction, two-tailed p-value).

Supplemental Figure 2

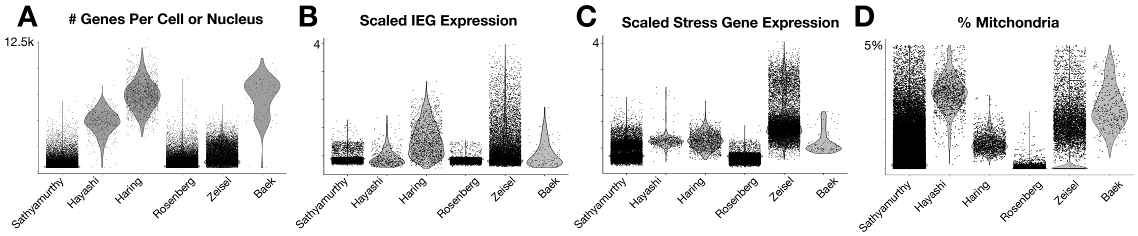

E

	Sathyamurthy	Hayashi	Haring	Rosenberg	Zeisel	Baek
**OPC**	322	3	8	950	790	
**Oligo Progen-1**	59	2	10	706	582	1
**Oligo Progen-2**	167		25	469	629	
**Oligos-1**	3822	5	118	943	2832	3
**Oligos-2**	807		30	470	922	
**Schwann**	1165			9	1	
**Peripheral glia/ NC derivative**	298			2	1	
**Meninges-1**	1661	5	6	444	18	
**Meninges-2**	641		6	111		
**Ependymal**	468	2	52	260	249	
**Astrocytes-1**	2219	6	13	1457	1907	2
**Astrocytes-2**	443	3	1	694	309	
**Endothelial**	1075	3	15	393	1462	
**Pericytes**	463		9	310	826	
**Microglia**	466	9	17	438	640	16
**Neurons**	5473	421	1042	11201	1130	86
**TOTAL**	**19549**	**459**	**1352**	**18857**	**12298**	**108**

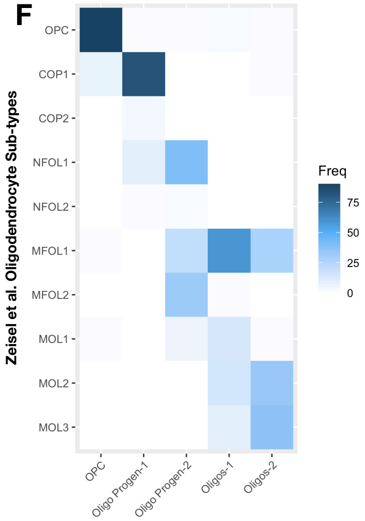

Supplemental Figure 2 (supplement for Main Figure 1). Integrated analysis of six independent studies to reveal spinal cord coarse cell types. (A-D) The cells/nuclei from each study varied in terms of the number of genes detected per cell/nucleus (A) (scale is 12,500), the expression of immediate early genes (IEG) (B) and stress related genes (C) (scale is 4 for both and the data are presented as the scaled expression of the gene module normalized to 100 randomly selected genes), and the percent of mitochondria (D). (E) Table presenting the number of cells/nuclei from each study that were present in each of the coarse cell type clusters. Low-quality nuclei from the Sathyamurthy dataset are included, though they were discarded as such or labeled as neurons in preliminary analysis but discarded later in the neuron sub-analysis. (F) Label transfer using the full Zeisel oligodendrocyte lineage data as a reference (rows) confirmed harmonized cluster annotation (columns). The frequency of cells/nuclei from each harmonized cluster being annotated with a given Zeisel label is shown, normalized by column.

Supplemental Figure 3

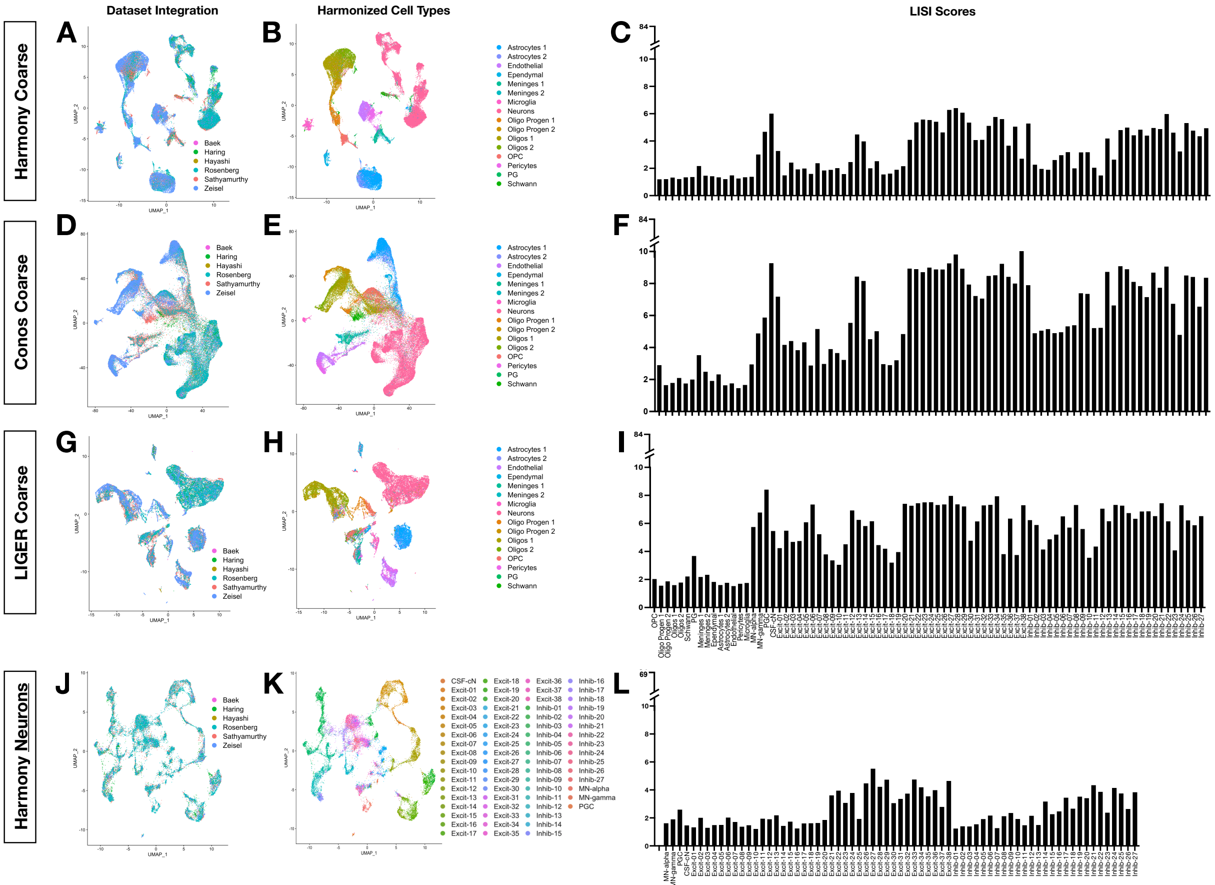

Supplemental Figure 3 (supplement to Main Figure 1). Comparison of three independent integration methodologies. (A-I) Harmony, Conos, and LIGER integration was performed on the merged dataset of all cell types from six independent studies and are presented in their own UMAP space colored by dataset (A,D,G) or colored by the coarse cell types obtained from Seurat integration (B,E,H). Local Inverse Simpson Index (LISI) values were used to assess the diversity of cells within local neighborhoods in integrated UMAP space with the definitions of cell types based on Seurat integration (C,F,I). Out of the 84 possible cell types, most cells showed very homogenous local neighborhoods based on low LISI scores. (J,K,L) A similar analysis was repeated on neurons only, using Harmony as the integration method and is shown in a UMAP colored by dataset (J) or the Seurat-integrated neuronal populations (K) and as analyzed by LISI for the 69 possible neuronal cell types (L).

Supplemental Figure 4

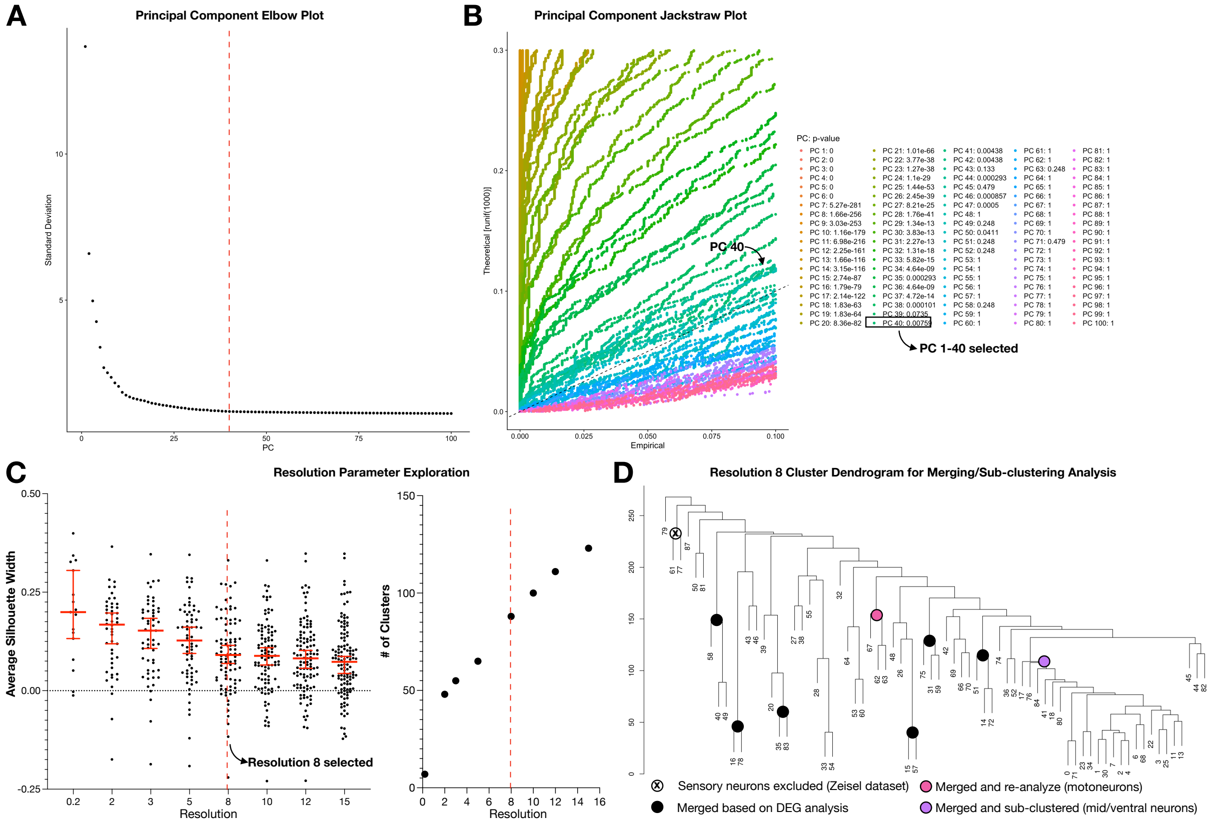

Supplemental Figure 4 (supplement to Main Figure 2). Parameter selection for neuronal clustering. (A,B) 40 principal components were used to analyze the neuronal cell types, a parameter that was selected based on being within the asymptotic range in the elbow plot (A), being just above statistical significance in the jackstraw plot (B), and inspection of the gene loadings for each principal component. PC = principal component.
(C) Resolution 8 was selected to analyze the neuronal cell types based on being in a stable range of cluster robustness (left, determined by average silhouette width) and comparison of generated cluster marker genes with known markers and co-expression patterns from the literature. This generated an intermediate number of cluster (right). For each resolution tested, a different number of clusters were generated, each shown as a single point, with the mean +/- standard error. (D) Dendrogram of cluster relationships (using 4000 integrated genes hclust. Average) that was used to identify pairs of related clusters as candidates for merging (see Methods for more detail). Clusters 61 and 77 represented sensory neurons from the Zeisel dataset and were excluded. Black dots indicate clusters that were merged. Pink dot indicates motoneuron clusters that were merged and sub-clustered in their own principal component space. Purple dot indicates mid/ventral clusters that were merged and sub-clustered in their own principal component space (see Methods for more detail).

Supplemental Figure 5

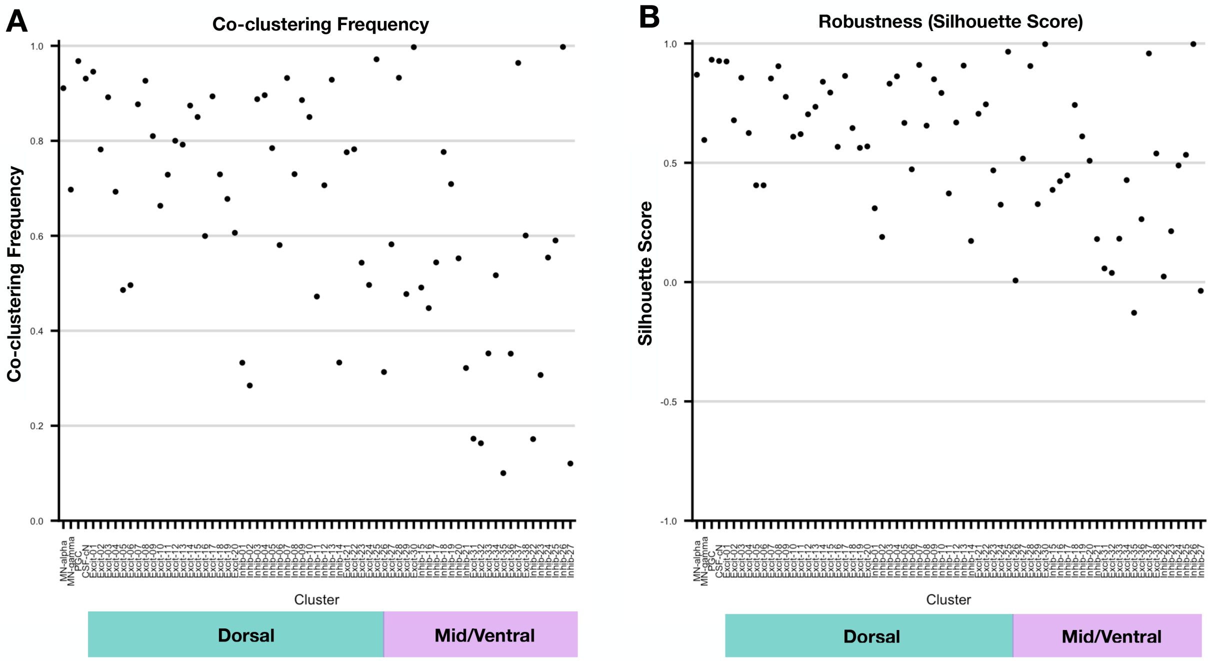

Supplemental Figure 5 (supplement to Main Figure 2). Integrated analysis of six independent studies to define 69 spinal cord neuron cell types of varying robustness. (A) The co-clustering frequency of the cells/nuclei from each cluster when clustering was automated and run 100 times using a random 80% of the dataset each time, analyzed in two tiers (first tier: mid/ventral grouped together; second tier: mid/ventral sub-types only). (B) The "robustness score" (the silhouette value of the co-clustering frequency matrix) of each cluster is shown.

Supplemental Figure 6

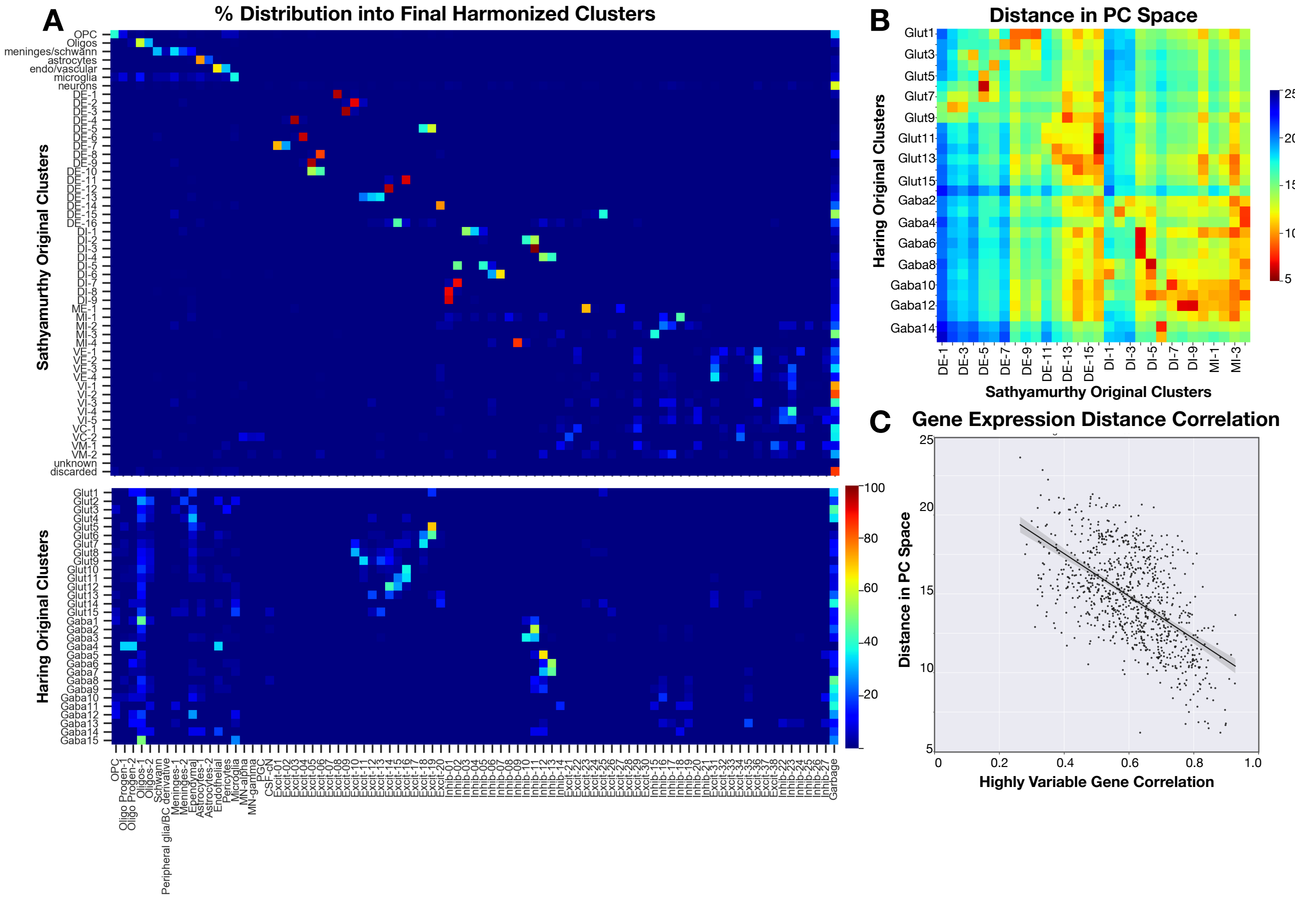

Supplemental Figure 6 (supplement to Main Figure 2). Relationship with the Sathyamurthy et al. and Haring et al. spinal cord atlases. (A) The distribution of cells from the original clusters of the Sathyamurthy and Haring datasets (rows) into the harmonized clusters (columns), ranging from 0 blue to 100% red distribution. (B) The distance between the centroids of the cells/nuclei from the original Haring and Sathyamurthy clusters, measured in 50 dimensional principal component (PC) space. Only dorsal neuron clusters are shown for the Sathyamurthy dataset and in both datasets, every other cluster is labeled. Distance is colored from short (red) to long (blue). (C) Relationship between the distance in PC space and the correlation in gene expression each pair of clusters between the Haring and Sathyamurthy datasets, with each point represent the distance for one pair (not replicates). Linear regression was used to create a best fit line, shown with 95% confidence interval.

Supplemental Figure 7

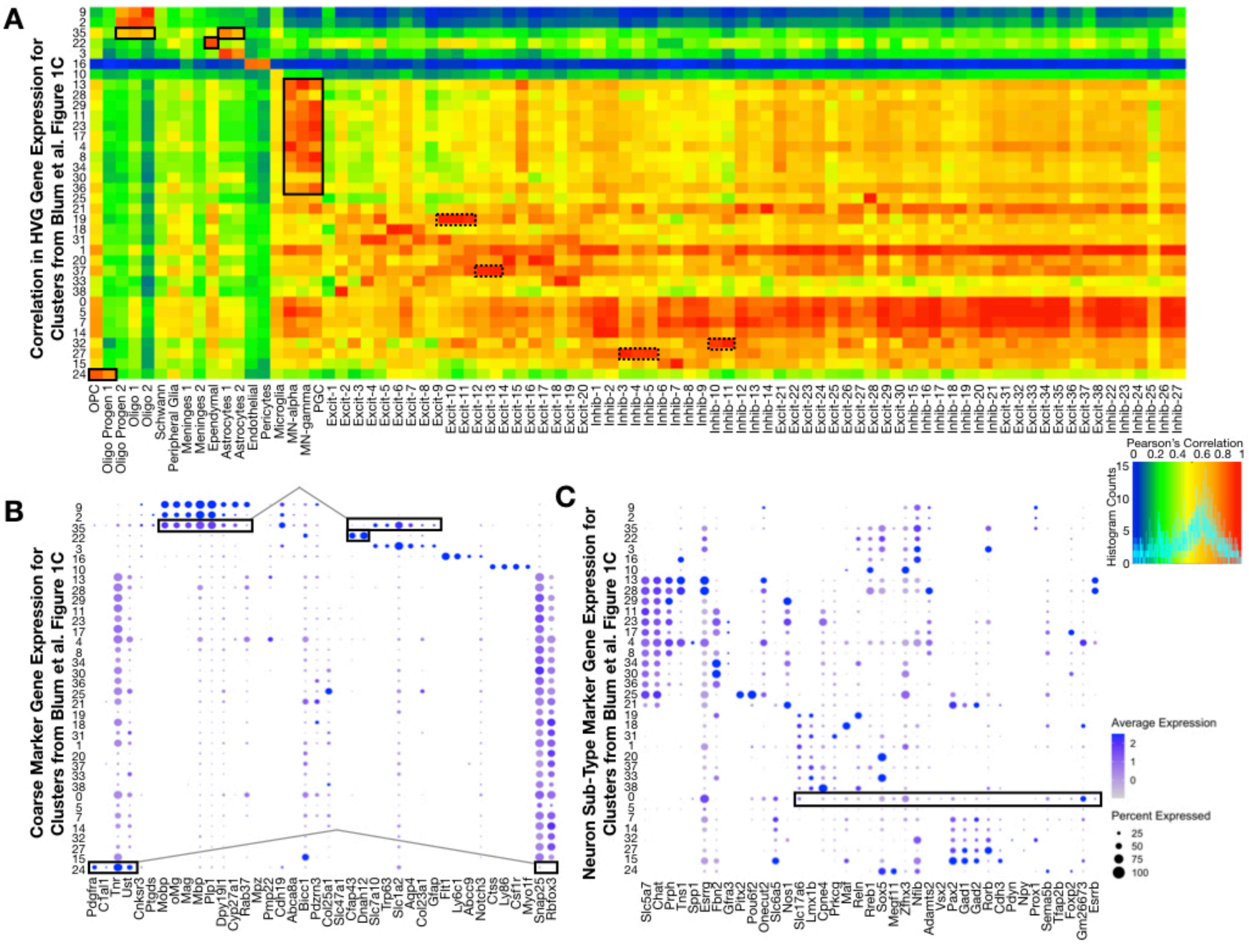

Supplemental Figure 7 (supplement to Main Figure 2): Relationship with the Blum et al. spinal cord atlas. (A) Correlation in average gene expression between each cluster from Blum et al. (rows) and each harmonized cluster (columns), using the top 500 highly variable genes. Pearson's correlation score is presented from 0 (blue) to 1 (red). Black solid line boxes surround datapoints with notable differences. Cluster 35 shows similar correlations with oligodendrocyte precursors and astrocytes. Cluster 22 shows a strong correlation with ependymal cells. Clusters 13-36 represent a greater diversity of motoneurons than MN-alpha, MN-gamma, and PGC. Cluster 24 shows a correlation with oligodendrocyte precursor cells. Black dashed line boxes surround examples of individual Blum et al. clusters that show a correlation to multiple harmonized clusters. (B,C) Dot plots showing harmonized atlas marker gene expression as expressed in the Blum et al. clusters, including coarse cell type markers (B) or neuron sub-types and neuron family markers (C). Dot color intensity reflects average expression level and dot size reflects the percent of nuclei in each cluster expressing each marker. The boxes in (B) reflect the hybrid/mixed/doublet gene expression of oligodendrocyte and astrocyte markers in cluster 35, the expression of ependymal marker genes in cluster 22, and the expression of oligodendrocyte precursor/progenitor markers and the lack of expression of general neural markers in cluster 24. The box in (C) reflects the broad marker gene expression in cluster 0 including of inhibitory markers Pax2, Gad1, and Gad2 and the excitatory markers Slc17a6 and Vsx2.

Supplemental Figure 8

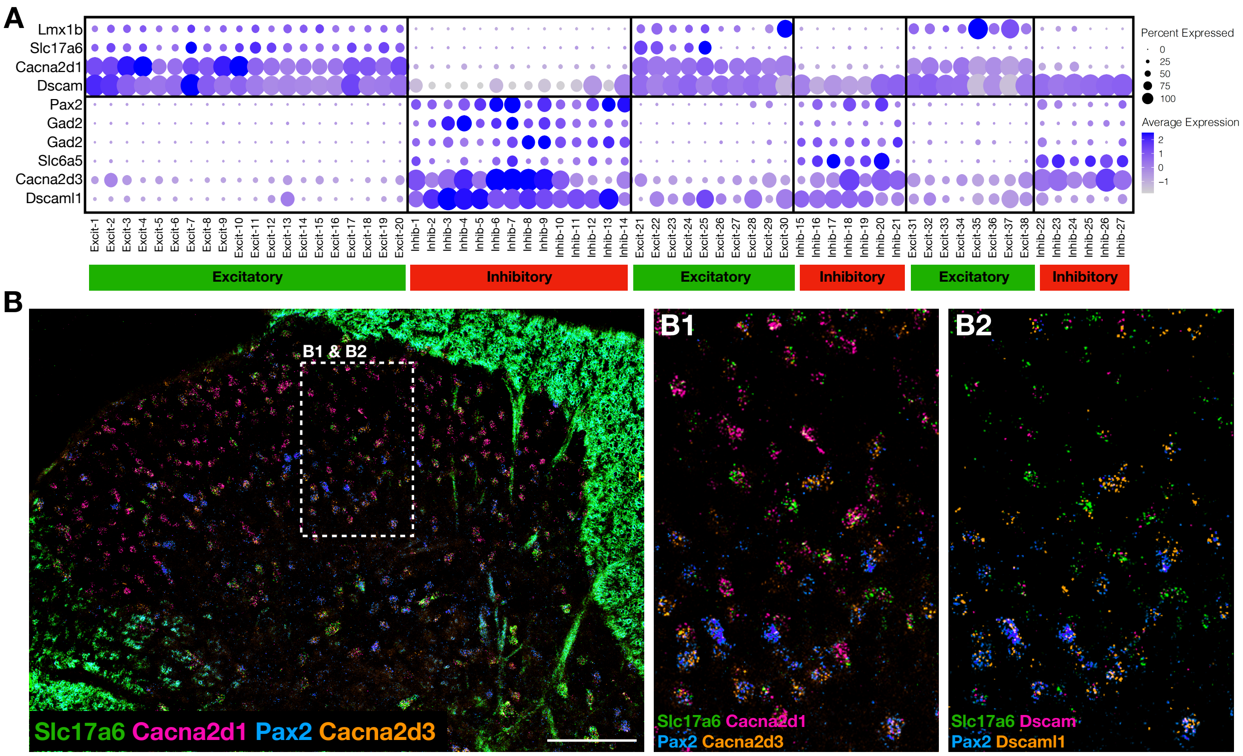

Supplemental Figure 8 (supplement to Table 1): Trends in excitatory-inhibitory organization of spinal cord neuron types. (A) Dot plot showing expression of genes associated with neurotransmitter class. (B) Validation of differentially expressed genes by RNA *in situ* hybridization. 20x tiled images, with brightness and contrast adjusted. All images are representative of the pattern observed in at least 3 sections each from N=3 animals. Scale bar is 100 µm.

Supplemental Figure 9

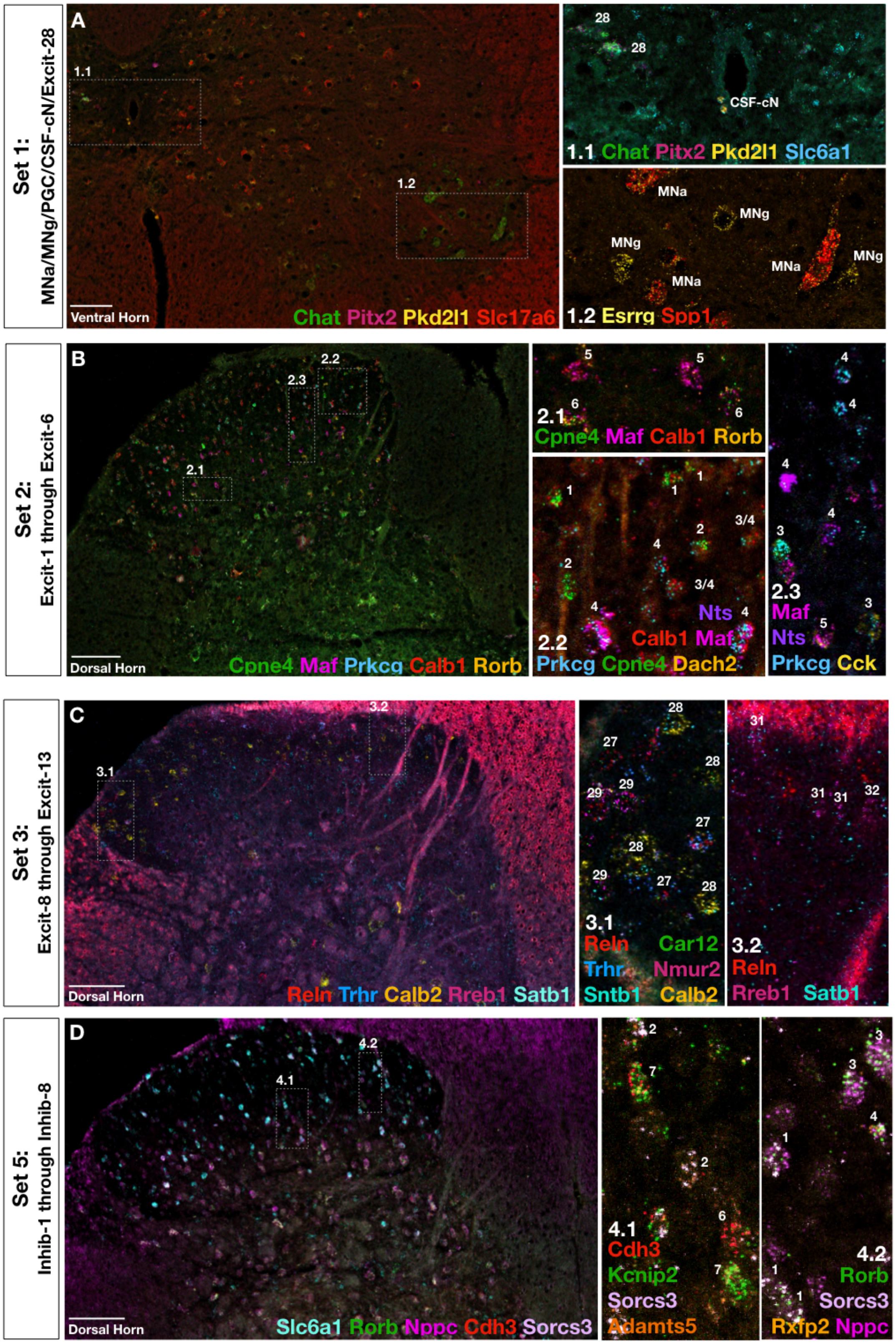

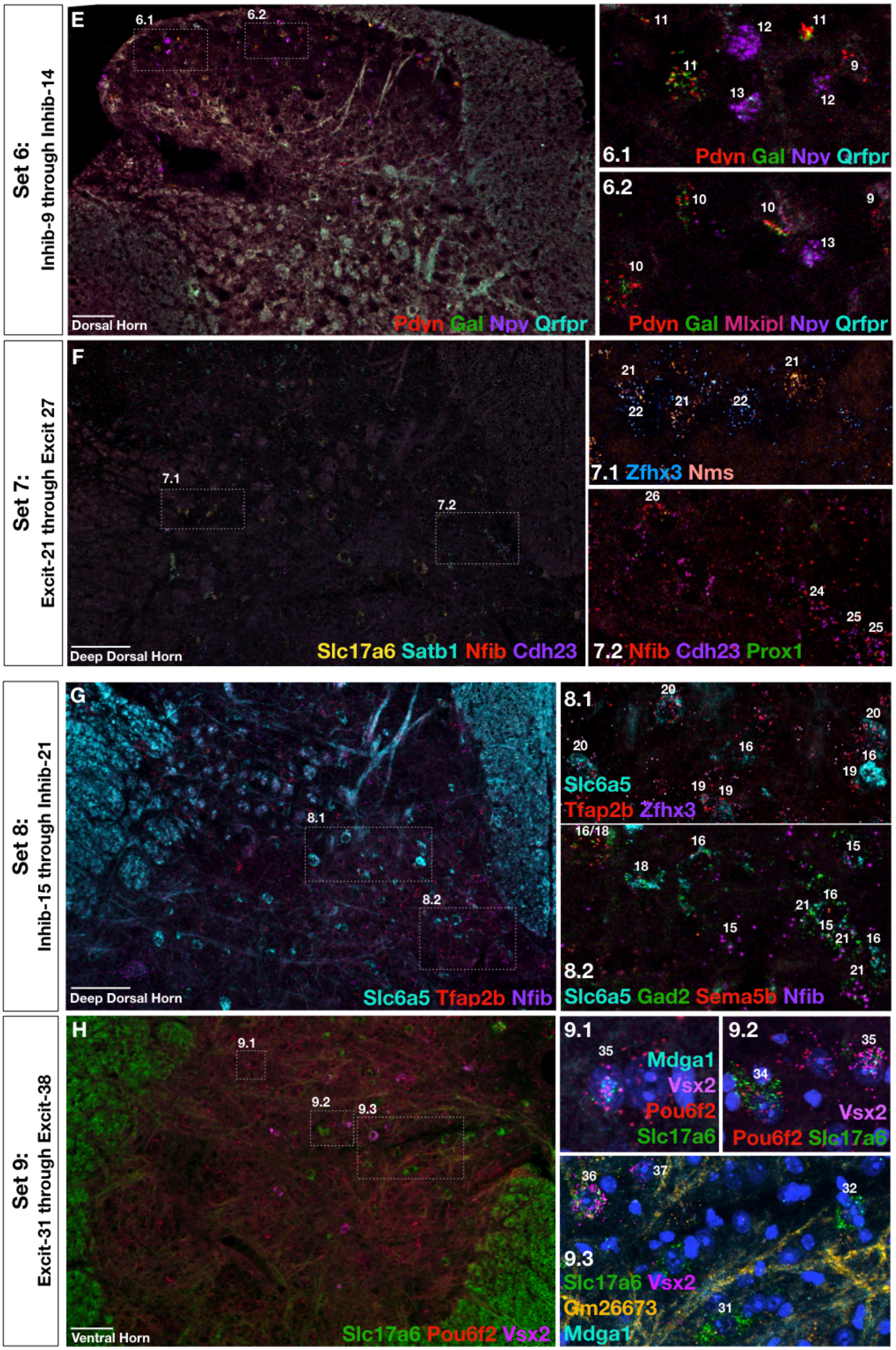

Set 6:
Inhib-9 through Inhib-14
E
6.1
6.2
Dorsal Horn
Pdyn Gal Npy Qrfpr
6.1 Pdyn Gal Npy Qrfpr
6.2 Pdyn Gal Mlxipl Npy Qrfpr
Set 7:
Excit-21 through Excit 27
F
7.1
7.2
Deep Dorsal Horn
Slc17a6 Satb1 Nfib Cdh23
7.1 Zfhx3 Nms
7.2 Nfib Cdh23 Prox1
Set 8:
Inhib-15 through Inhib-21
G
8.1
8.2
Deep Dorsal Horn
Slc6a5 Tfap2b Nfib
8.1 Slc6a5 Tfap2b Zfhx3
8.2 Slc6a5 Gad2 Sema5b Nfib
Set 9:
Excit-31 through Excit-38
H
9.1
9.2
9.3
Ventral Horn
Slc17a6 Pou6f2 Vsx2
9.1 Mdga1 Vsx2 Pou6f2 Slc17a6
9.2 Vsx2 Pou6f2 Slc17a6
9.3 Slc17a6 Vsx2 Gm26673 Mdga1

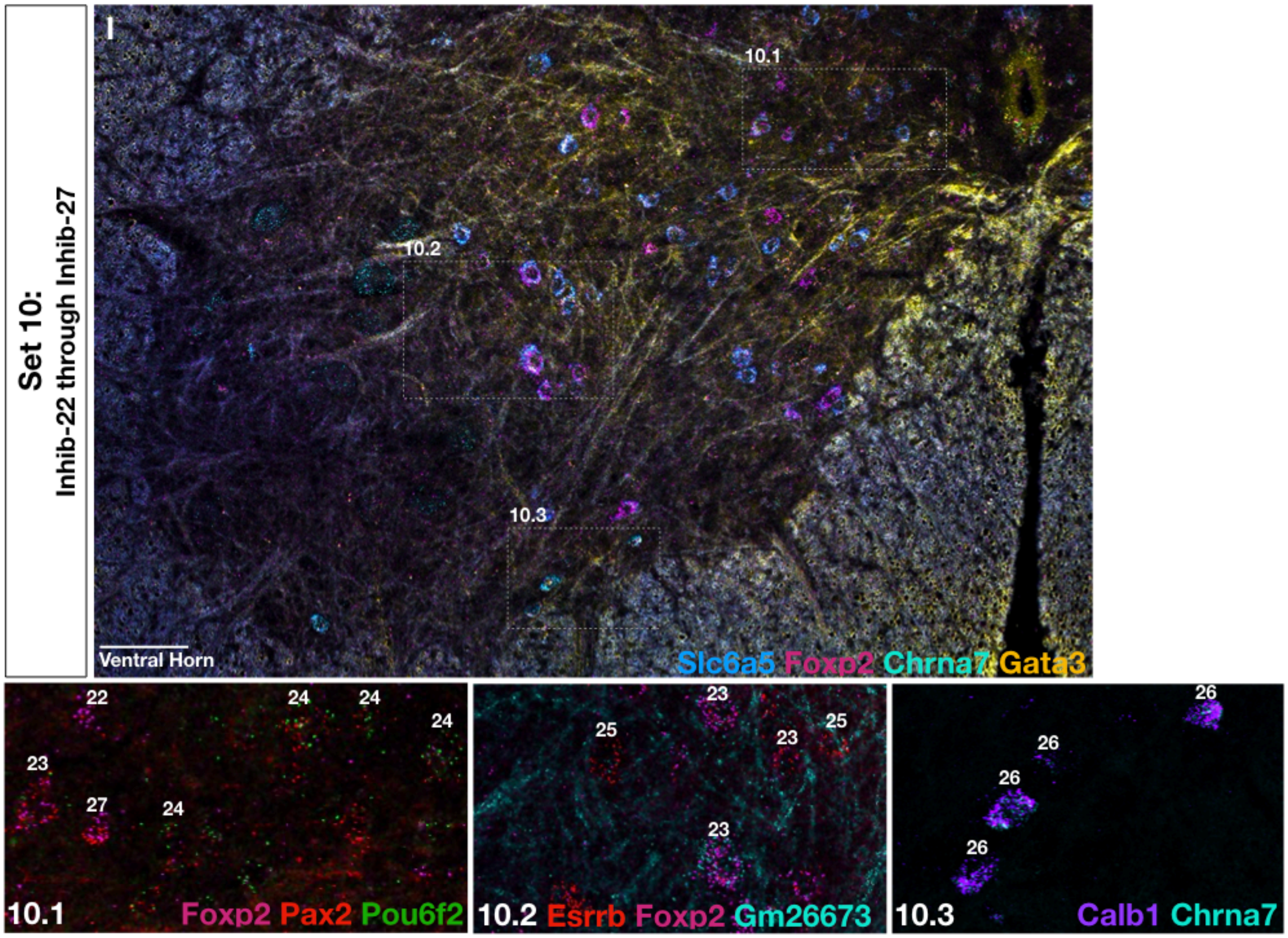

Supplemental Figure 9 (supplement to Main Figure 4). Multi-plexed RNA in situ hybridization of a combinatorial panel of spinal cord cell type marker genes. For each of the ten sets of RNAScope probes (listed in Supplemental Table 3), this figure shows a 20x tiled image, as well as multiple higher magnification images that are boxed in the 20x tiled image and labeled by Set#.Inset# names. The expressed genes are shown for each image and the cell-type identity is shown by small white numbers next to positive cells in the inset pictures. 20x tiled images, with brightness and contrast adjusted. All images are representative of the pattern observed in at least 3 sections each from N=3 animals. Scale bars are 100 μm.

Supplemental Figure 10

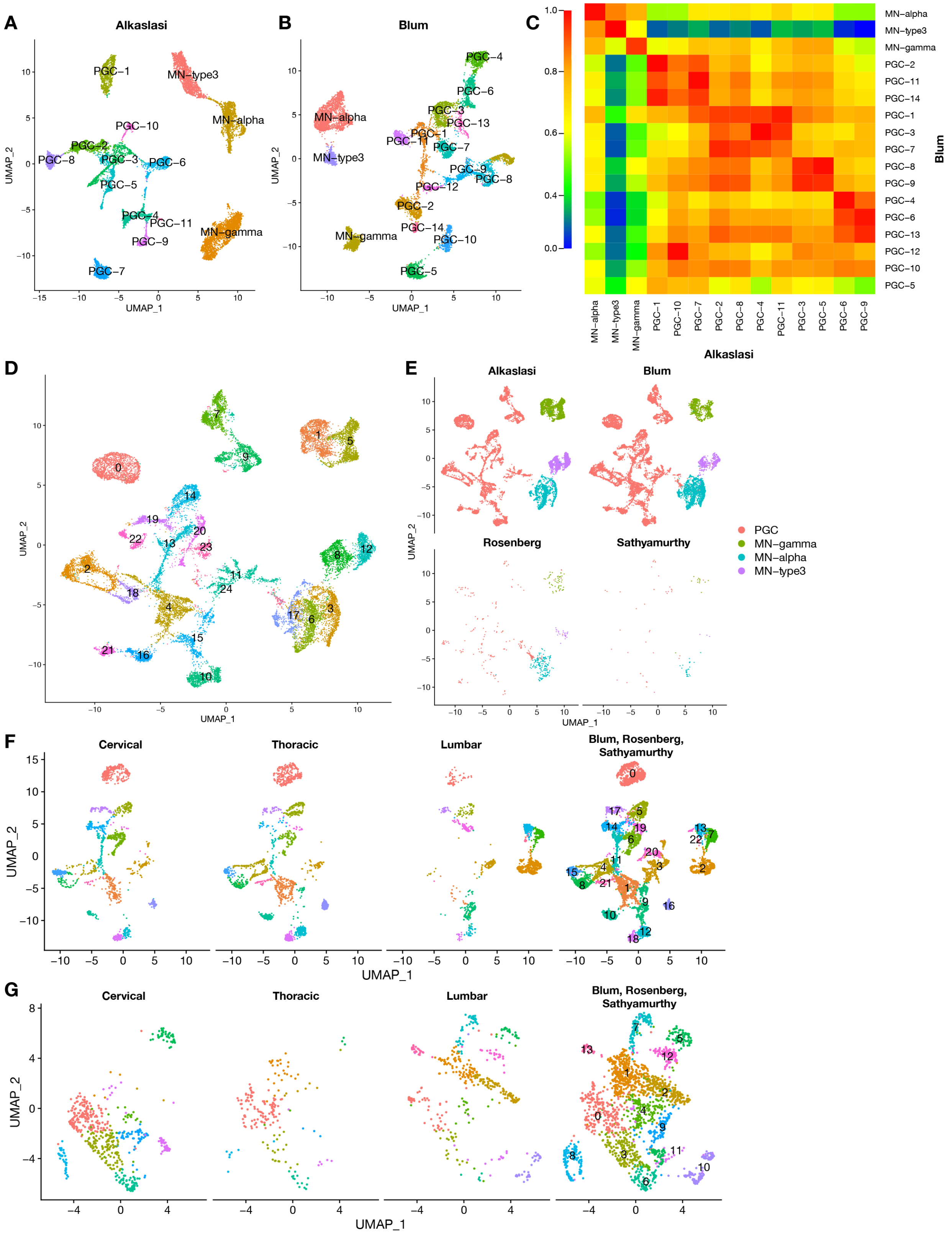

Supplemental Figure 10 (supplement to Table 1). Focused analysis of spinal motoneuron diversity from four independent datasets reveals previously unrecognized diversity and enables spatial mapping of neuronal subtypes. (A) UMAP representation of motor neurons from the Alkaslasi dataset, highlighting 3 types of skeletal motor neurons and 11 preganglionic motor neurons. (B) UMAP representation of motor neurons from the Blum dataset, highlighting 3 types of skeletal motor neurons and 14 preganglionic motor neurons. (C) Correlation between Alkaslasi and Blum clusters, showing strong correspondence of clusters between datasets. (D) Integration of 23,032 single neurons from 4 independent datasets increases resolution of motor neuron clustering, resulting in 25 clusters. (E) UMAPs of integrated datasets, with nuclei/neurons split into their source datasets and colored by cell type. (F, G) Integrated clustering of PGCs and of alpha motor neurons increases resolution of clustering. Combined with spatial barcoding of Alkaslasi dataset, this resolution enables the identification of distinctly localized subtypes that were not previously resolved. (F) PGCs are clustered into 23 subtypes that vary by spinal cord level. The integrated data resolves a lumbar-only cluster that was not previously recognized (2). (G) Alpha motor neurons are clustered into 14 subtypes that vary by spinal cord level. While the Alkaslasi data alone revealed primarily cervical clusters, it did not reveal lumbar-only clusters. The integrated data highlights the lumbar-only clusters 7 and 10. Further, the combined datasets reveal that the digit-innervating motor neurons, expressing *Cpne4* and *Fign*, segregate into 2 subtypes, one in both cervical and lumbar (cluster 5), and one confined to lumbar spinal cord (cluster 12).

Supplemental Figure 11

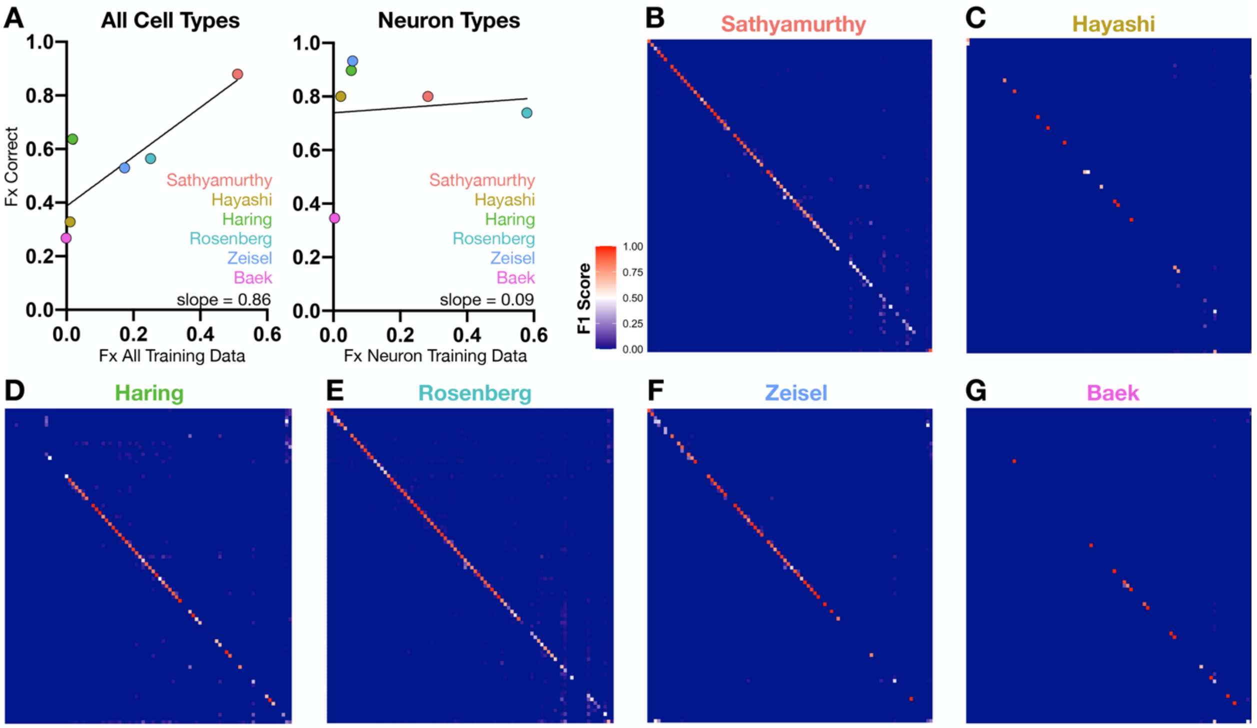

Supplemental Figure 11 (supplement to Main Figure 6). Performance of the two-tiered model on each independent dataset. (A) The fraction of correctly predicted cells/nuclei using the two-tiered label transfer and neural network model on each independent dataset, plotted against the fraction of the training dataset from each independent dataset. Overall data is on the left and the neuron-specific data is on the right. Simple linear regression was performed to determine the slope of the relationship between the fraction of each dataset that was correct and the fraction of the training data represented by that dataset. (B-G) Heatmaps of the F1 scores for each cell type in each study, colored from blue=0 to red=1. The actual cell types are in rows and the predicted cell types are in columns, both in the order presented in Table 1 and Figure 5E, with “doublets” and “junk” in the final two rows/columns. * denotes that, for the Sathyamurthy dataset, a random sub-sample of 20% of the data was used to generate the data in this figure because the full dataset was too large to process through label transfer.

Supplemental Figure 12

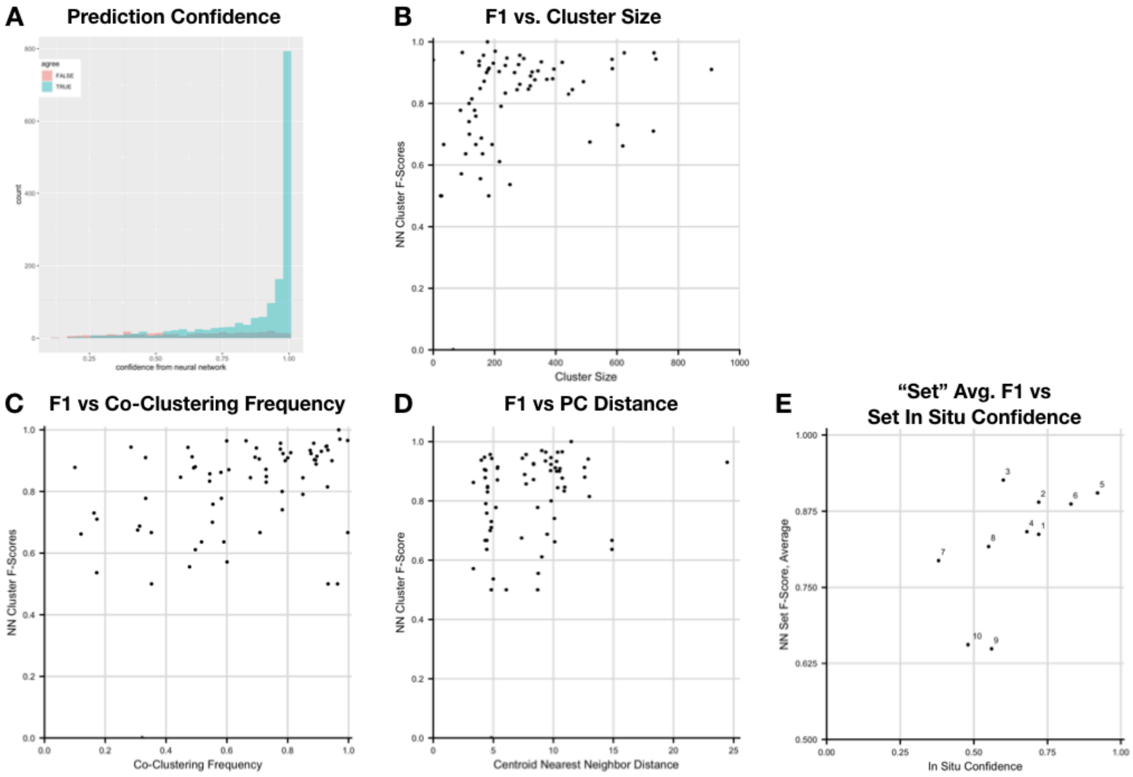

Supplemental Figure 12 (supplement to Main Figure 6). Neural network performance and comparison to cluster robustness. (A) The confidence (x-axis) with which each cell/nucleus was classified (counts, y-axis), colored by whether the prediction was correct (blue, true) or incorrect (false, pink). (B) Scatterplot of the neural network F1 score and the size of each cluster. (C) Scatterplot of the neural network F1 score and the co-clustering frequency of each cluster. (D) Scatterplot of the neural network F1 score and the distance between the centroid of each cluster and the centroid of its nearest neighbor in 50-dimensional principal component (PC) space. (E) Scatterplot of the neural network F1 score (presented as an average for each "set" of clusters) and the percent of cells in that set that could be confidently assigned to a single cluster by in situ hybridization analysis.

Supplemental Figure 13

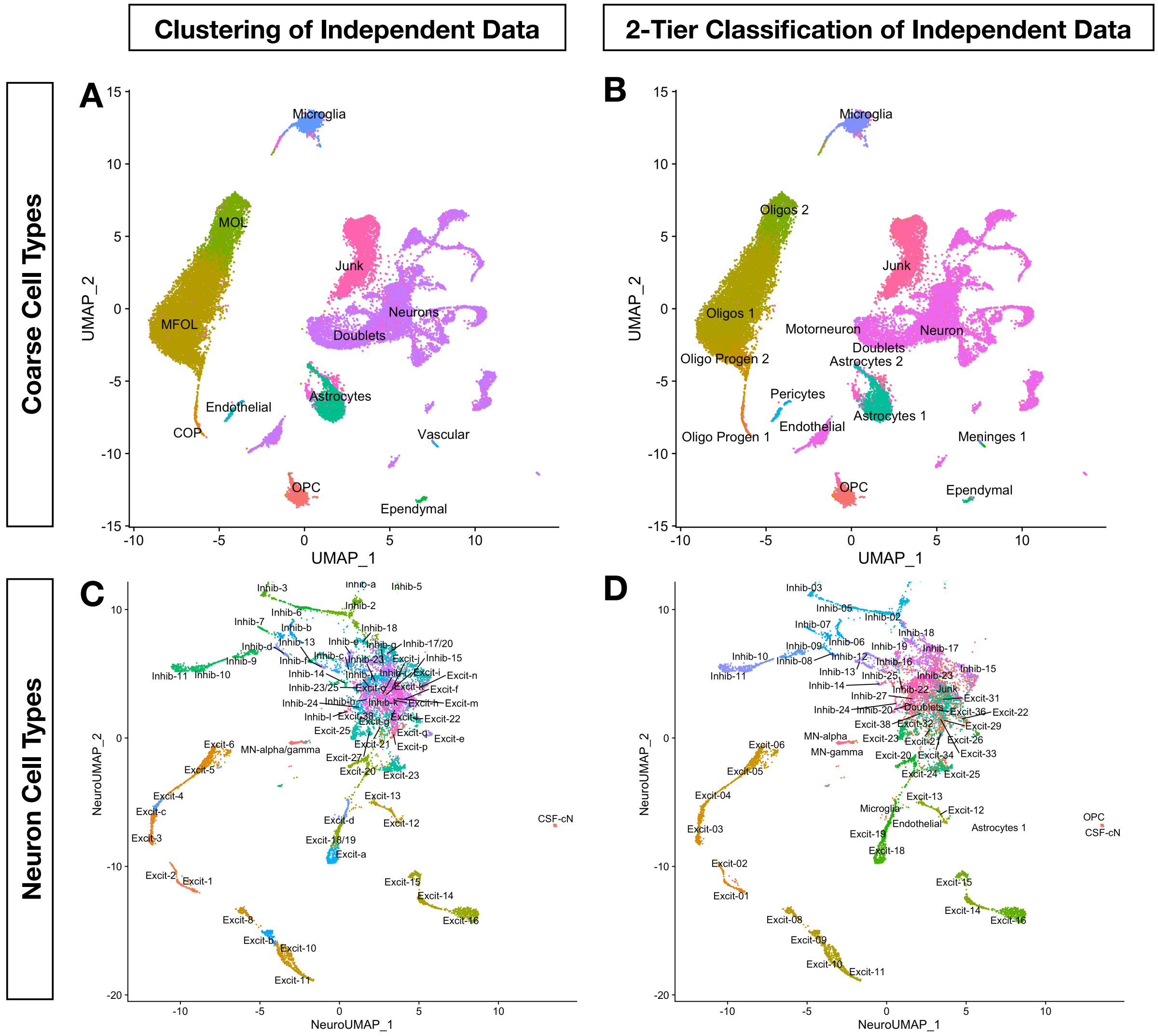

Supplemental Figure 13 (supplement to Main Figure 6). Comparison of clustering and automated classification of the independent dataset. (A) UMAP plot showing the coarse cell type of each nucleus based on clustering of the independent dataset. (B) UMAP plot showing the coarse cell type of each nucleus based on based on two-tiered computational classification. (C) UMAP plot showing the neuron type of each nucleus based on clustering of the independent dataset. Clusters that could not be identified at a fine resolution were given placeholder names such as "Inhib-b". (D) UMAP plot showing the neuron cell type of each nucleus based on based on two-tiered computational classification.
