# Supplementary material for: A harmonized atlas of mouse spinal cord cell types and their spatial organization
Source: Nat Commun. 2021 Sep 29;12:5722. doi: 10.1038/s41467-021-25125-1 (PMC8481483; doi:10.1038/s41467-021-25125-1)
Supplement: Supplementary file 2 — Description of Additional Supplementary Files [file 41467_2021_25125_MOESM2_ESM.docx]

Description of Additional Supplementary Files

**Title: Supplementary Movie 1.**

Description: Supplementary file, rotating video file of the three-dimensional UMAP reduction for the harmonized neuronal sub-populations.
