# Supplementary figures and images for: A harmonized atlas of mouse spinal cord cell types and their spatial organization
Source: Nat Commun. 2021 Sep 29;12:5722. doi: 10.1038/s41467-021-25125-1 (PMC8481483; doi:10.1038/s41467-021-25125-1)

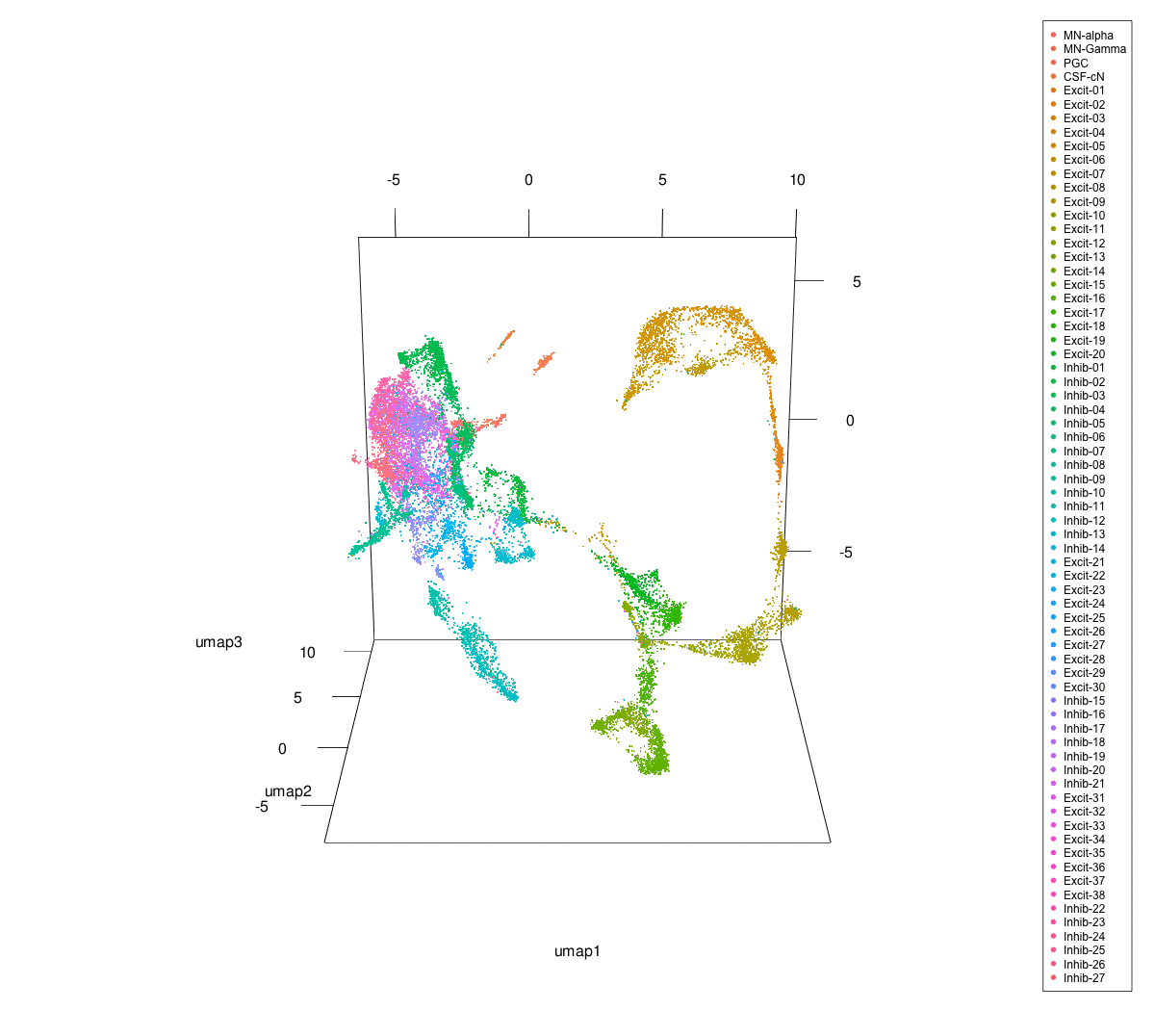

Supplement: Supplementary file 3 — Supplementary Movie 1 [file 41467_2021_25125_MOESM3_ESM.gif]
