# Supplementary material for: A harmonized atlas of mouse spinal cord cell types and their spatial organization
Source: Nat Commun. 2021 Sep 29;12:5722. doi: 10.1038/s41467-021-25125-1 (PMC8481483; doi:10.1038/s41467-021-25125-1)
Supplement: Supplementary file 4 — Reporting Summary [file 41467_2021_25125_MOESM4_ESM.pdf]

nature research

Corresponding author(s): Ariel J. Levine

Last updated by author(s): 09 JULY 2021

Reporting Summary

Nature Research wishes to improve the reproducibility of the work that we publish. This form provides structure for consistency and transparency in reporting. For further information on Nature Research policies, see our Editorial Policies and the Editorial Policy Checklist.

Statistics

For all statistical analyses, confirm that the following items are present in the figure legend, table legend, main text, or Methods section.

n/a	Confirmed	
☐	☒	The exact sample size (n) for each experimental group/condition, given as a discrete number and unit of measurement
☐	☒	A statement on whether measurements were taken from distinct samples or whether the same sample was measured repeatedly
☐	☒	The statistical test(s) used AND whether they are one- or two-sided<br>*Only common tests should be described solely by name; describe more complex techniques in the Methods section.*
☐	☒	A description of all covariates tested
☐	☒	A description of any assumptions or corrections, such as tests of normality and adjustment for multiple comparisons
☐	☒	A full description of the statistical parameters including central tendency (e.g. means) or other basic estimates (e.g. regression coefficient) AND variation (e.g. standard deviation) or associated estimates of uncertainty (e.g. confidence intervals)
☐	☒	For null hypothesis testing, the test statistic (e.g. F, t, r) with confidence intervals, effect sizes, degrees of freedom and P value noted<br>*Give P values as exact values whenever suitable.*
☒	☐	For Bayesian analysis, information on the choice of priors and Markov chain Monte Carlo settings
☐	☒	For hierarchical and complex designs, identification of the appropriate level for tests and full reporting of outcomes
☐	☒	Estimates of effect sizes (e.g. Cohen's d, Pearson's r), indicating how they were calculated

Our web collection on statistics for biologists contains articles on many of the points above.

Software and code

Policy information about availability of computer code

Data collection	All data were downloaded from public repositories at the identifiers listed in the "Data" section below
Data analysis	Python, v3.8.5<br>numpy, v1.19.1<br>pandas, v1.1.0<br>scikit-learn, v0.22.2.post1<br>scipy, v1.5.2<br>tensorflow, v2.2 - v2.4<br>R, v3.5.0 - v4.1.2<br>Seurat, v3<br>tidyverse, v1.3.0<br>lineup, v0.37-11<br>CellRanger, v3.1<br>Picard, v2.18.20

For manuscripts utilizing custom algorithms or software that are central to the research but not yet described in published literature, software must be made available to editors and reviewers. We strongly encourage code deposition in a community repository (e.g. GitHub). See the Nature Research guidelines for submitting code & software for further information.

Data

Policy information about availability of data

All manuscripts must include a data availability statement. This statement should provide the following information, where applicable:

- Accession codes, unique identifiers, or web links for publicly available datasets
- A list of figures that have associated raw data
- A description of any restrictions on data availability

The raw sequencing data generated in this study have been deposited in the NCBI database under accession code GSE158380 (https://www.ncbi.nlm.nih.gov/geo/query/acc.cgi?acc=GSE158380). In addition, the publicly available data utilized in this study are available at:
Sathyamurthy: https://www.ncbi.nlm.nih.gov/geo/query/acc.cgi?acc=GSE103892
Hayashi: https://www.ncbi.nlm.nih.gov/geo/query/acc.cgi?acc=GSE108788
Zeisel: https://www.ncbi.nlm.nih.gov/sra/SRP135960
Haring: https://www.ncbi.nlm.nih.gov/geo/query/acc.cgi?acc= GSE103840
Rosenberg: https://www.ncbi.nlm.nih.gov/geo/query/acc.cgi?acc=GSE110823
Baek: https://www.ncbi.nlm.nih.gov/geo/query/acc.cgi?acc=GSE130312
Blum: https://www.ncbi.nlm.nih.gov/geo/query/acc.cgi?acc=GSE161621
Alkaslasi: https://www.ncbi.nlm.nih.gov/geo/query/acc.cgi?acc=GSE167597
Delile: https://www.ebi.ac.uk/arrayexpress/experiments/E-MTAB-7320/files)

A searchable version of all processed data from the harmonized analysis is available www.seqseek.ninds.nih.gov.

Field-specific reporting

Please select the one below that is the best fit for your research. If you are not sure, read the appropriate sections before making your selection.

[x] Life sciences [ ] Behavioural & social sciences [ ] Ecological, evolutionary & environmental sciences

For a reference copy of the document with all sections, see nature.com/documents/nr-reporting-summary-flat.pdf

Life sciences study design

All studies must disclose on these points even when the disclosure is negative.

Sample size	For animal studies, an even balance of male and female mice that were 9 weeks old and of mixed C57BL/6J and BALB/cJ background were used for single nucleus sequencing (four mice) and validation studies (two groups of three mice), with sample sizes being equal to or greater than commonly accepted standards in the field.
Data exclusions	We analyzed all cells and nuclei with at least 200 detected genes (to exclude low quality or "empty" barcodes) and with less than 5% of transcripts being mitochondrial (to exclude lysing cells or mitochondria-nuclei doublets).
Replication	All RNA in situ hybridization, immunofluorescence, and WFA lectin staining experiments and associated quantification were performed on at least three independent animals, with data from individual replicates and the mean presented (ex. in Figure 3). For replication of the two-tier classification approach, an entirely independent dataset was used. All other computer analyses were deterministic.
Randomization	As all mice were of wild-type genetic background, and there were no treatment groups, randomization was not used in this study.
Blinding	As all mice were of wild-type genetic background, and there were no treatment groups, blinding was not used in this study.

Reporting for specific materials, systems and methods

We require information from authors about some types of materials, experimental systems and methods used in many studies. Here, indicate whether each material, system or method listed is relevant to your study. If you are not sure if a list item applies to your research, read the appropriate section before selecting a response.

Materials & experimental systems

n/a	Involved in the study	
☐	☒	Antibodies
☒	☐	Eukaryotic cell lines
☒	☐	Palaeontology and archaeology
☐	☒	Animals and other organisms
☒	☐	Human research participants
☒	☐	Clinical data
☒	☐	Dual use research of concern

Methods

n/a	Involved in the study	
☒	☐	ChIP-seq
☒	☐	Flow cytometry
☒	☐	MRI-based neuroimaging

Antibodies

Antibodies used	Camk2a Millipore 905-532<br>WFA lectin: Vector Laboratories FL-1351-2
Validation	To detect CAMK2, Millipore 905-532 was used (1:500). For perineuronal net WFA-lectin staining, fluorescent lectin (Vector Laboratories, FL-1351-2) was used according to the manufacturer's instructions. Both Camk2a and WFA-lectin expression patterns matched previously published work (ex. Galtrey...Fawcett et al. 2008).

Animals and other organisms

Policy information about studies involving animals; ARRIVE guidelines recommended for reporting animal research

Laboratory animals	An even balance of male and female mice that were 9 weeks old and of mixed C57BL/6J and BALB/cJ background were used for single nucleus sequencing (four mice) and validation studies (two groups of three mice). Mice were housed in a standard 12-hr light dark cycle and at standard temperature and humidity.
Wild animals	This is not relevant to the study.
Field-collected samples	This is not relevant to the study.
Ethics oversight	Animal experiments were performed in accordance with institutional guidelines and approved (protocol #1384) by the National Institute of Neurological Disorder and Stroke's Institutional Animal Care and Use Committee.

Note that full information on the approval of the study protocol must also be provided in the manuscript.
